# EGFR tyrosine kinase activity and Rab GTPases coordinate EGFR trafficking to regulate macrophage activation in sepsis

**DOI:** 10.1038/s41419-022-05370-y

**Published:** 2022-11-07

**Authors:** Xuedi Zhang, Cuiping Chen, Chunxiu Ling, Shuhua Luo, Ziying Xiong, Xiaolei Liu, Chaoxiong Liao, Pengyun Xie, Youtan Liu, Liangqing Zhang, Zhanghui Chen, Zhifeng Liu, Jing Tang

**Affiliations:** 1 The Department of Anesthesiology, Affiliated Hospital of Guangdong Medical University, Zhanjiang, 524000 Guangdong China; 2 Guangdong Medical University, Zhanjiang, 524000 Guangdong China; 3 The Department of Anesthesiology, Shenzhen Hospital, Southern Medical University, Shenzhen, 518000 Guangdong China; 4 Department of Hematology, Zhanjiang Institute of Clinical Medicine, Zhanjiang Central Hospital, 524000 Zhanjiang, China; 5 The Department of Critical Care Medicine, General Hospital of Southern Theater Command of PLA, Guangzhou, 510010 Guangdong China

**Keywords:** Toll-like receptors, Bacterial infection

## Abstract

EGFR phosphorylation is required for TLR4-mediated macrophage activation during sepsis. However, whether and how intracellular EGFR is transported during endotoxemia have largely been unknown. Here, we show that LPS promotes high levels cell surface expression of EGFR in macrophages through two different transport mechanisms. On one hand, Rab10 is required for EEA1-mediated the membrane translocation of EGFR from the Golgi. On the other hand, EGFR phosphorylation prevents its endocytosis in a kinase activity-dependent manner. Erlotinib, an EGFR tyrosine kinase inhibitor, significantly reduced membrane EGFR expression in LPS-activated macrophage. Mechanistically, upon LPS induced TLR4/EGFR phosphorylation, MAPK14 phosphorylated Rab7a at S72 impaired membrane receptor late endocytosis, which maintains EGFR membrane localization though blocking its lysosomal degradation. Meanwhile, Rab5a is also involved in the early endocytosis of EGFR. Subsequently, inhibition of EGFR phosphorylation switches M1 phenotype to M2 phenotype and alleviates sepsis-induced acute lung injury. Mechanistic study demonstrated that Erlotinib suppressed glycolysis-dependent M1 polarization via PKM2/HIF-1ɑ pathway and promoted M2 polarization through up-regulating PPARγ induced glutamine metabolism. Collectively, our data elucidated a more in-depth mechanism of macrophages activation, and provided stronger evidence supporting EGFR as a potential therapeutic target for the treatment of sepsis.

## Graphical abstract

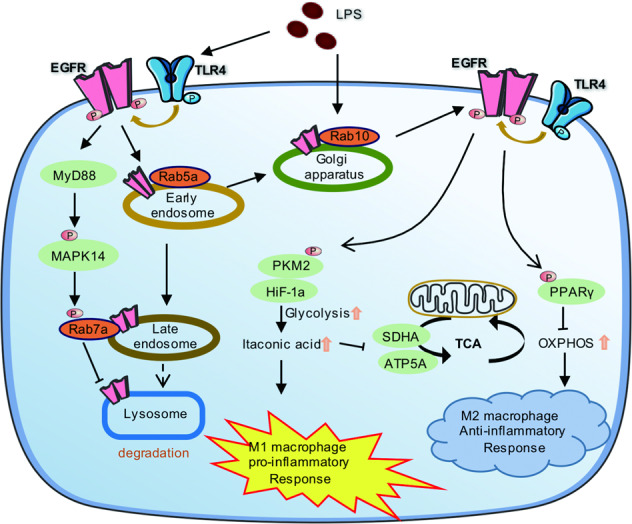

## Introduction

Sepsis refers to tissue damage and even life-threatening multiple organ dysfunction syndrome caused by the maladjustment of the body’s innate immune response to pathogen infection [1, 2]. As regarded its pathogenesis, continued activation of neutrophils, monocytes and macrophages may attribute to accelerate the septic response [3].

Metabolic cascades are increasingly recognized as characteristics and controllers of macrophage activation. M1 macrophages (classically activated macrophages) obtain energy for rapid killing through glycolysis, whereas M2 macrophages (alternatively activated macrophages) rely on mitochondrial oxidative phosphorylation (OXPHOS) for energy supplying [4, 5]. M1 macrophages are pro-inflammatory and have a central role in host defense against infection, while M2 macrophages are associated with responses to anti-inflammatory reactions and tissue repair [6, 7]. The balance between the macrophage M1/M2 phenotype is critical for controlling excessive inflammation and triggering wound healing. Therefore, regulating macrophage polarization emerges is a potential therapeutic approach for effective treatment of inflammatory diseases such as sepsis.

EGFR (epidermal growth factor receptor) is a transmembrane receptor tyrosine kinase which also has been reported to play an important role in modulating LPS/TLR4 signaling [8, 9]. Both we and other researchers reported that EGFR inhibitor Erlotinib effectively prevents LPS-induced cytokine expression in vivo, and protects mice from LPS-induced lethality suggesting a cross-talk between TLR4 and EGFR signaling pathways, which importantly affects the host prognosis following bacterial infection [10–12]. Our lab further demonstrated that EGFR and TLR4 co-regulate macrophage activation in endotoxemia and EGFR phosphorylation is necessary to increase TLR4 cell surface expression and signal transduction [13]. However, compared with TLR4, little is known about the process and physiological significance of intracellular transportation of EGFR in macrophage in response to LPS.

In this study, we found LPS increases the expression of EGFR on the cell surface of macrophages and Rab10 helps plasma membrane transport of EGFR. We also showed that Rab5a mediates early EGFR endocytosis, while EGFR/MAPK14/Rab7a regulates late EGFR endocytosis and lysosomal degradation pathway. We further demonstrated that inhibition of cell surface EGFR expression decreases glycolysis-dependent M1 polarization and promotes M2 polarization through activating PPARγ-mediated glutamine metabolism. Finally, inhibition of EGFR phosphorylation skewed the balance of macrophages from M1 to M2 phenotype and blunted LPS-induced inflammation and tissue injury. Altogether, our data elucidated a more in-depth mechanism of macrophages activation, and provided stronger evidence supporting EGFR as a potential therapeutic target for the treatment of sepsis.

## Results

### LPS promotes cell surface expression of EGFR in macrophages

**Fig. 1:**
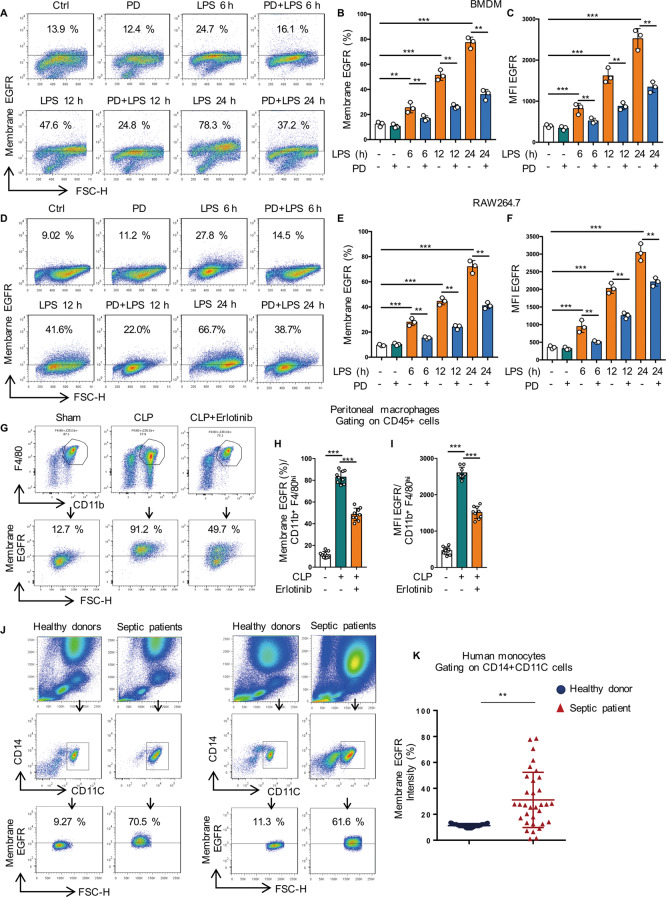
LPS promotes cell surface expression of EGFR in macrophage. **A**–**F** Macrophages were treated with LPS (1 µg/mL) for 6, 12, or 24 h with or without PD168393 (PD, 10 µM)) pretreatment for 30 min. **A** Flow cytometry analysis of EGFR cell surface expression in BMDM. **B** Percentage of EGFR-positive macrophage is shown. **C** Mean fluorescence intensity (MFI) is shown (*n* = 3). **D** Flow cytometry analysis of EGFR cell surface expression in RAW264.7. **E** Percentage of EGFR-positive macrophage is shown. **F** Mean fluorescence intensity (MFI) is shown (*n* = 3). **G**–**I** Macrophages were collected from peritoneal lavage of C57BL/6 mice subjected to CLP and were divided into Sham-operated, CLP and CLP plus Erlotinib (100 mg/kg, gavage) pretreatmend for 2 h, and peritoneal macrophages were identified with CD45 + CD11b + F4/80high. **G** EGFR intensity on the surface of peritoneal macrophage was analyzed by flow cytometry. **H** Percentage of EGFR-positive peritoneal macrophage is shown (*n* = 9). **I** Mean fluorescence intensity (MFI) is shown (*n* = 9). **J**, **K** Blood was collected from patients with clinical sepsis or health donors, and blood mononuclear cells were identified with CD14 and CD11c. **J** EGFR intensity on the surface of Mononuclear cells were analyzed by flow cytometry (20 Healthy donors and 33 Septic patients). **K** Percentage of EGFR-positive CD14 + CD11c + mononuclear cell is shown. The graphs depict mean ± SD based on three independent experiments. *P < 0.05, **P < 0.01, ***P < 0.001.

We have reported that LPS increased the cell surface expression of TLR4 and inhibition of EGFR phosphorylation decreased TLR4 cell surface expression in response to LPS [13]. Here we demonstrated that LPS also improved the cell surface expression of EGFR in both BMDM (Fig. 1A–C) and RAW264.7 cells (Fig. 1D–F) and this phenomenon could be effectively inhibited by EGFR inhibitor PD168393 at all time points. To further convince the role of EGFR cell surface expression in inflammation macrophages, a cecal ligation and puncture (CLP)-induced sepsis mouse model was performed as described previously [14].

Then we detected the expression of EGFR in the peritoneal macrophages of C57BL/6 mice following CLP for 24 h with or without Erlotinib (100 mg/kg gavage administration) pretreatment for 2 h. EGFR expression was increased to 5-fold in peritoneal macrophages after 24 h post CLP compared with Sham group, but Erlotinib pretreatment inhibited the increase of EGFR expression on the cell surface of peritoneal macrophages of C57BL/6 mice in response to CLP procedure (Fig. 1G–I). More importantly, in most clinical acute septic patients (Supplementary Table 6), EGFR expression on the mononuclear cell surface was obviously higher than that of healthy volunteers (Fig. 1J, K). Given that EGFR can be activated by both ligand-dependent and -independent mechanisms, in vitro studies were next conducted to address the role of EGF in the LPS/TLR4/EGFR signaling. BMDM cells were pretreated with the neutralizing anti-EGF antibody to eliminate the effect of any EGF in an autocrine manner, followed by LPS-treatment. We found that neutralizing anti-EGF antibody pretreatment did not change the total protein level of EGFR (Fig. S4A–F). Given this data, we concluded that LPS-induced macrophage M1/M2 polarization is EGFR ligand-independent. All these results indicated that LPS induces the activation of EGFR and promotes the expression of EGFR on the cell surface of macrophage in endotoxemia and sepsis.

### Phosphorylation of Rab7a on S72 promotes late EGFR endocytosis

Inhibition of EGFR phosphorylation significantly reduced EGFR expression in macrophage membrane, suggesting the important role of EGFR kinase activity in receptor transport. To uncover novel downstream targets of EGFR kinase activity, quantitative label-free LC-MS/MS proteomic was performed to quantify the whole cell lysate in RAW264.7 cells. In total, 8151 peptide fractions were analyzed, yielding 11772 unique phosphosites mapping to 3041 unique proteins (Fig. S1A). Phosphorylated peptides mainly enriched in MAPK signaling, endocytosis and glycolysis pathway with KEGG pathway analysis (Fig. S1B). Among the identified differentially expressed phosphopeptides, only Rab7a is reported to be involved in Endocytosis pathway. Rab7a GTPase is involved in regulating endocytosis-mediated protein trafficking [15, 16]. In particular, Rab7A facilitates trafficking of membrane receptors, such as growth factor receptor, from early endosome to late endosome and lysosome for their ultimate degradation [17]. Therefore, we focused on the relation between RAB7a phosphorylation and EGFR trafficking.

**Fig. 2:**
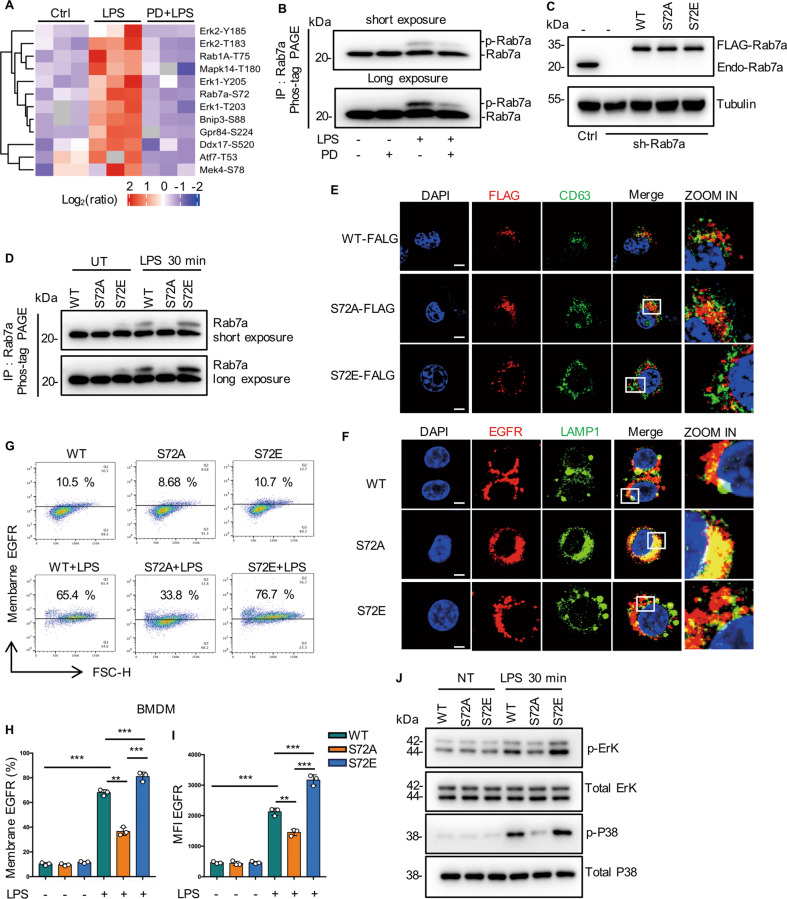
Phosphorylation of Rab7a on S72 promotes late EGFR endocytosis. **A** Cluster analysis of differentially expressed proteins and phosphopeptides in RAW264.7 measured after treated with LPS (1 µg/mL) for 30 min with or without PD168393 (PD 10 µM) pretreatment for 30 min, when compared with control. Blue and red indicates down-or upregulation, respectively (*n* = 3 samples of each condition). **B** PD168393 (PD 10 µM) inhibited Rab7a phosphorylation in response to LPS (30 min). The indicated cells were subjected to depolarization, and cell extracts were subjected to phostag-PAGE with Rab7a antibody by immunoblotting. **C** Stable expression of Rab7a-WT, Rab7a-S72A or Rab7a-S72E mutant in Rab7a shRNA knockdown RAW264.7 cells. Rab7a total protein expression was measure by western blot. a-Tubulin as a loading control. **D** a-Rab7a immunoprecipitates from the indicated cells were separated by phostag-PAGE followed by immunoblotting with a-Rab7a antibodies. **E** Immune-staining of Flag-Rab7a and CD63 in the indicated Rab7a mutant RAW264.7 cells followed by LPS treatment (1 µg/mL) for 30 min. Scale bar, 5 μm. **F** Immune-staining of EGFR and LAMP1 in the indicated Rab7a mutant RAW264.7 cells followed by LPS treatment (1 µg/mL) for 24 h. Scale bar, 5 μm. **G**–**I** The indicated Rab7a mutant RAW264.7 cells followed by LPS treatment (1 µg/mL) for 24 h. **G** EGFR expression on the surface of RAW264.7 was analyzed by flow cytometry. **H** Percentage of EGFR-positive RAW264.7 is shown (*n* = 3). **I** Mean fluorescence intensity (MFI) is shown (*n* = 3). **J** Cell lysates from the indicated Rab7a mutant RAW264.7 cells followed by LPS treatment (1 µg/mL) for 24 h were prepared, and phospho-ERK, total ERK, total p38 and phospho-p38 protein expression was measure by western blot. The graphs depict mean ± SD based on three independent experiments. *P < 0.05, **P < 0.01, ***P < 0.001.

In particular, only one phosphorylation site, S72 was found in Rab7a among the differentially phosphorylated sites (Fig. 2A; Fig. S1C and S1D). With phosphate-affinity (phos-tag) polyacrylamide gel electrophoresis (PAGE), we successfully detected Rab7a phosphorylation in RAW264.7 cells at 30 min after LPS treatment (Fig. 2B, lane 3), and the phosphorylation could inhibit by PD168393 (Fig. 2B, lane 4). To further confirm S72 as the major phospho-site of Rab7a, we knocked down Rab7a in RAW264.7 cells and re-expressed Rab7a-WT, the non-phosphorylatable S72A mutant and the potential phosphomimetic S72E mutant using lentivirus at near endogenous level (Fig. 2C). In contrast to Rab7a-WT, Rab7a-S72E appeared one more pronounced band shift in the phos-tag PAGE analysis (Fig. 2D, lane 4, lane 6), while Rab7a-S72A did not (Fig. 2D, lane 5). These results suggested that Rab7a is phosphorylated at the S72 site in LPS-activated macrophages.

Some studies have reported that phosphorylation of S72 resulted in complete loss of the GTPase activity of Rab7a [18, 19], suggesting that the key role of Rab7a phosphorylation in the intracellular transport of EGFR. Compared with Rab7a-WT, the colocalization increased between Rab7a-S72A and CD63 (late endosome), but decreased between Rab7a-S72E and CD63 in RAW264.7 cells stimulated by LPS for 30 min (Fig. 2E). In contrast to Rab7a-S72E mutant, the colocalization between EGFR and LAMP1 increased and cell surface expression of EGFR decreased (Fig. 2G–I), indicating that the late endocytic degradation of EGFR was enhanced after treated with LPS for 12 h in Rab7a-S72A mutant (Fig. 2F). In addition, we observed that Rab7a-S72E mutant promoted ERK1/2 and P38 activation, but not in Rab7a-S72A mutant (Fig. 2J). All these results indicated that Rab7a phosphorylation regulates the late endocytic degradation of EGFR and thus affects the membrane expression of EGFR.

### MAPK14 phosphorylates Rab7a at S72 site

**Fig. 3:**
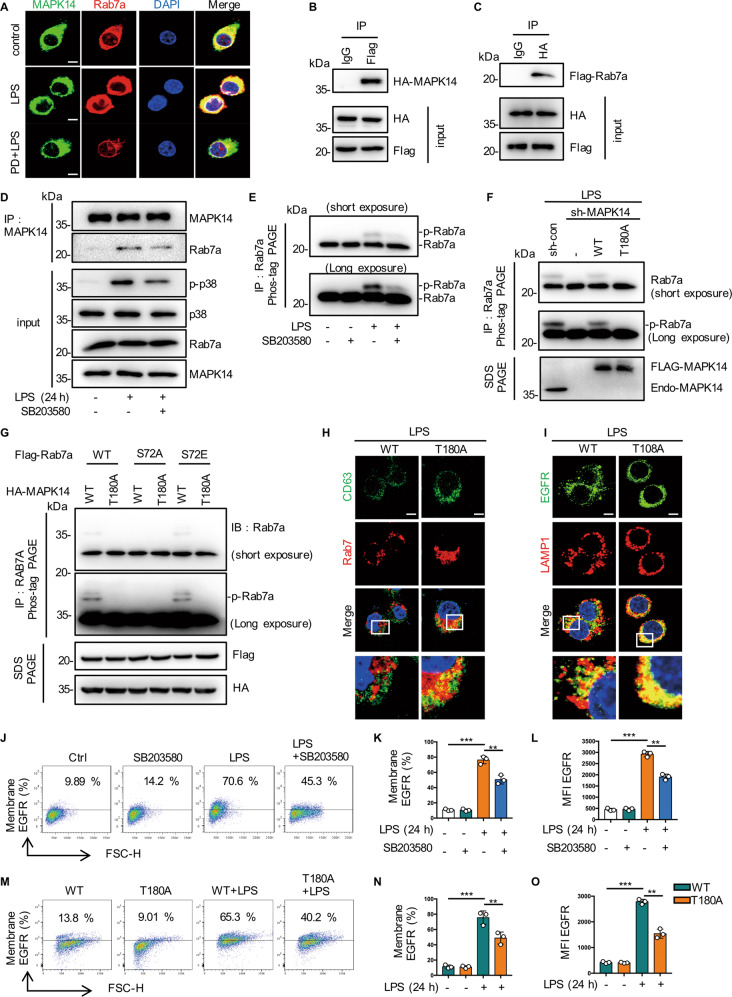
MAPK14 phosphorylates Rab7a at S72 site. **A** Immune-staining of MAPK14 and Rab7a in RAW264.7 treated with 1 µg/mL LPS for 30 min, with or without PD168393 (PD 10 μM) pretreatment for 30 min. **B**, **C** Co-immunoprecipitation between Flag-tagged Rab7a and HA-tagged MAPK14 in HEK 293 T cells transfected with the Flag-tagged Rab7a and HA-tagged MAPK14 construct. **D** Immunoblot analysis of MAPK14 immunoprecipitates of lysates of RAW264.7 stimulated with LPS (1 μg/mL) for 30 min with or without PD168393 (PD) pretreatment for 30 min. **E** Immunoblot analysis of Rab7a immunoprecipitates of lysates of RAW264.7 stimulated with LPS (1 μg/mL) for 30 min with or without p38 inhibitor SB-203580 (SB 5 μM) pretreatment for 30 min. **F** Immunoblot analysis of Rab7a immunoprecipitates of lysates of MAPK14 mutant RAW264.7 cells were stimulated with LPS (1 μg/mL) for 30 min, and cell extracts were subjected to a-Rab7a immunoprecipitation before phostag-PAGE. **G** MAPK14 induces phosphorylation of Rab7 at S72 in vivo. HEK293 cells were co-transfected with Flag–Rab7 (WT, S72A or S72E mutants) and HA–MAPK14 (WT or T180A inactivation mutant), and cell lysates were analyzed by phos-tag SDS-PAGE. Total lysates were immunoblotted (IB) with antibodies as indicated. The migrating bands indicate phosphorylated Rab7, respectively. **H** Immune-staining of Rab7a and CD63 in the indicated MAPK14 mutant RAW264.7 cells followed by LPS treatment (1 μg/mL) for 30 min. Scale bar, 5 μm. **I** Immune-staining of EGFR and LAMP1 in the indicated MAPK14 mutant RAW264.7 cells followed by LPS treatment (1 μg/mL) for 24 h. Scale bar, 5 μm. **J**–**L** RAW264.7 cells were stimulated with LPS for 24 h with or without p38 inhibitor SB203580 (SB 5 μM). **J** EGFR expression on the surface of RAW264.7 was analyzed by flow cytometry. **K** Percentage of EGFR-positive RAW264.7 is shown (*n* = 3). **L** Mean fluorescence intensity (MFI) is shown (*n* = 3). **M**–**O** MAPK14 mutant RAW264.7 cells were stimulated with LPS for 24 h with or without p38 inhibitor SB203580 (SB 5 μM). **M** EGFR expression on the surface of RAW264.7 was analyzed by flow cytometry (*n* = 3). **N** Percentage of EGFR-positive RAW264.7 is shown (*n* = 3). **O** Mean fluorescence intensity (MFI) is shown (*n* = 3). The graphs depict mean ± SD based on three independent experiments. *P < 0.05, **P < 0.01, ***P < 0.001.

According to phosphorylated proteomic data, we hypothesized that the phosphorylation of Rab7a at S72 may be regulated by the EGFR/MAPK14 pathway. First we found the increased colocalization of Rab7a with MAPK14 in LPS treated RAW264.7 cells, but reduced by PD168393 pretreatment (Fig. 3A). Then flag-tagged MAPK14 co-precipitated with HA-tagged Rab7a in the various constructs co-transfected HEK 293 T cells (Fig. 3B, C). MAPK14 inhibitor SB203580 could inhibit the endogenous binding of MAPK14 to Rab7a in RAW264.7 cells (Fig. 3D). In addition, SB203580 attenuated endogenous Rab7a phosphorylation in LPS-stimulated BMDMs with phos-tag PAGE analysis of cell extracts (Fig. 3E). Moreover, compared with MAPK14-WT (Fig. 3F, lane 1, lane 3), the inactivation mutation MAPK14-T180A did not appear a Rab7a protein mobility shift in LPS-activated RAW264.7 cells (Fig. 3F, lane 2, lane 4). These results indicated that MAPK14 can directly bind to and phosphorylate Rab7a. To confirm that Rab7a S72 is the major site of MAPK14 phosphorylation, we co-expressed various constructs of Rab7a and MAPK14 in HEK293T cells. We detected Rab7a proteins mobility shift bands when Rab7a WT or S72E mutant co-expressed with MAPK14-WT (Fig. 3G, lane 1, lane 5), but not with MAPK14-T180A (Fig. 3G, lane 2, lane 6). In addition, when co-expressed with Rab7a-S72A, MAPK14-WT and MAPK14-T180A showed no migrating band (Fig. 3G, lane 3, lane 4). All these results suggested that Rab7a S72 is the major site of MAPK14 phosphorylation in LPS-activated macrophages.

In order to understand the role of MAPK14 mediated Rab7a phosphorylation in the regulation of EGFR intracellular transport, we next analyzed the colocalization between Rab7a and CD63 in MAPK14 knock-down RAW264.7 cells with various MAPK14 constructs co-transfected. MAPK14-T180A mutant transfection enhanced the colocalization of Rab7a with CD63 30 min after LPS stimulation and reduced the colocalization of EGFR with LAMP1 24 h after LPS treatment (Fig. 3H, I). Therefore, we hypothesized that MAPK14/Rab7a might regulate membrane EGFR expression through an endosomal-lysosomal pathway. As expected, SB203580 significantly reduced LPS-induced EGFR expression in cell membrane (Fig. 3J–L). Meanwhile, the expression of membrane EGFR of MAPK14-T180A mutant was significantly decreased compared to the MAPK14-WT group (Fig. 3M–O). Altogether, these results indicated that MAPK14-mediated phosphorylation of Rab7a controls the late endocytosis and degradation of EGFR in LPS-activated macrophages.

### Rab5a mediates the early internalization of EGFR in macrophages

**Fig. 4:**
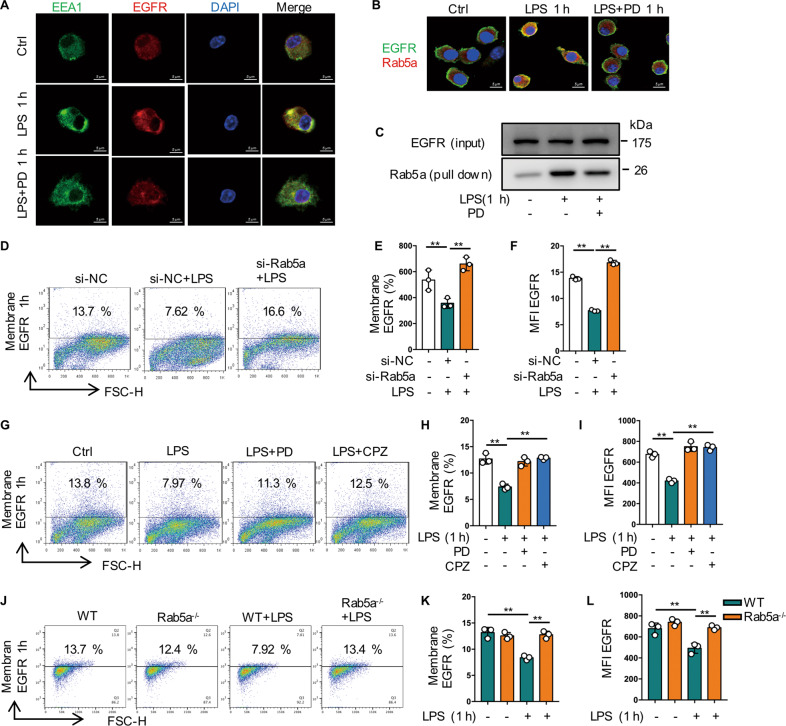
Rab5a mediates the early internalization of EGFR in macrophages. **A**–**C** BMDM were treated with LPS (1 µg/mL) for 1 h with or without PD168393 (PD 10 μM) pretreatment for 30 min. **A** Immune-staining of EGFR and EEA1 in BMDM. **B** Immune-staining of EGFR with Rab5a. **C** Co-immunoprecipitation between EGFR and Rab5a in BMDM. **D**–**F** BMDM transfected with si-NC or si-Rab5a for 48 h followed by LPS treatment (1 µg/mL) for 1 h. **D** EGFR expression on the surface of BMDM was analyzed by flow cytometry. **E** Percentage of EGFR-positive BMDM is shown (*n* = 3). **F** Mean fluorescence intensity (MFI) is shown (*n* = 3). **G**–**I** BMDM were treated with LPS (1 µg/mL) for 1 h, with or without clathrin inhibitor chlorpromazine (CPZ 12.5 μM) or PD168393 (PD 10 μM) pretreatment for 30 min. **G** EGFR expression on the surface of BMDM was analyzed by flow cytometry (*n* = 3). **H** Percentage of EGFR-positive BMDM is shown (*n* = 3). **I** Mean fluorescence intensity (MFI) is shown (*n* = 3). **J**–**L** WT and Rab5a^−/−^ BMDM were treated with LPS (1 μg/mL) for 1 h. **J** EGFR expression on the surface of BMDM was analyzed by flow cytometry (*n* = 3). **K** Percentage of EGFR-positive BMDM is shown (*n* = 3). **L** Mean fluorescence intensity (MFI) is shown (*n* = 3). The graphs depict mean ± SD based on three independent experiments. *P < 0.05, **P < 0.01, ***P < 0.001.

Endocytic trafficking of growth factor receptor is one of the vital cellular mechanisms for spatial and temporal regulation of EGFR signalling [20]. To more comprehensively investigate the mechanism of EGFR transport during macrophage inflammatory response, we sought to determine whether other small G-protein might be involved in EGFR trafficking. Here we found that PD168393 pretreatment attenuated the colocalization between early endosomes (EEA1) and EGFR at 1 h after LPS treatment (Fig. 4A). Since Rab5a is a key regulator of cellular endocytosis [21], we identified colocalization between Rab5a and EGFR near the plasma membrane of BMDM at 1 h after LPS treatment by confocal immunofluorescence microscopy and coimmunoprecipitation, which could be disturbed by PD168393 pretreatment (Fig. 4B, C). Knocking down of Rab5a prevented the decrease of EGFR expression on the cell surface of BMDM at 1 h after LPS treatment (Fig. 4D–F). Clathrin is the effector of Rab5a and involves in the internalization of various transmembrane receptors [22, 23]. Both PD168393 and clathrin inhibitor CPZ (Fig. 4G–I), also effectively suppressed the LPS-induced decrease of cell surface expression of EGFR at 1 h after treatment. Furthermore, in BMDM from Rab5a^−/−^ mice, compared with WT BMDM, Rab5a deficiency effectively prevented the decrease in the cell surface expression of EGFR at 1 h after LPS treatment (Fig. 4J–L). All these results indicate that LPS-induced internalization of EGFR is mediated by Rab5a.

### Rab10 helps surface expression of EGFR in macrophages

**Fig. 5:**
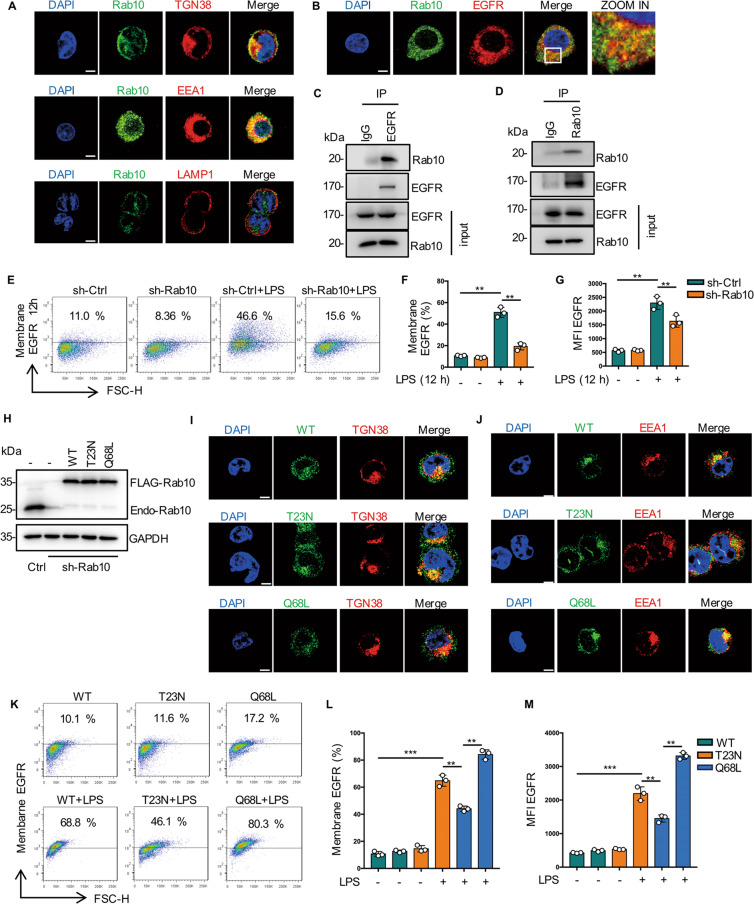
Rab10 helps surface expression of EGFR in macrophages. **A** EGFR is localized to Rab10-positive Golgi and early endosomes compartments. RAW2654.7 cells were immunostained with anti-TGN38K (red), anti-EEA1 (red), or anti-LAMP1 (red) and anti-Rab10 (green) Abs. Representative confocal images show colocalization (yellow) of Rab10 and TGN38K, EEA1, LAMP1. Scale bar, 5 μm. **B** Rab10 colocalized with EGFR in RAW264.7 cells. Scale bar, 5 μm. **C** Immunoblot analysis of EGFR immunoprecipitates of lysates of RAW264.7 cells. **D** Immunoblot analysis of Rab10 immunoprecipitates of lysates of RAW264.7 cells. **E**–**G** Sh-ctrl and sh-Rab10 BMDM were treated with LPS (1 μg/mL) for 12 h. **E** EGFR expression on the surface of BMDM was analyzed by flow cytometry. **F** Percentage of EGFR-positive BMDM is shown (*n* = 3). **G** Mean fluorescence intensity (MFI) is shown (*n* = 3). **H** Stable expression of Rab10-WT, Rab10-T23N or Rab10-Q68L mutant in Rab10 shRNA knockdown RAW264.7 cells. Immunoblotting of whole-cell extracts from the indicated cells using a-Rab10 or a-GAPDH as a loading control. **I** Immune-staining of TGN38 and indicated mutations of Rab10 in RAW264.7. **J** Immune-staining of EEA1 and indicated mutations of Rab10 in RAW264.7. **K**–**M** The indicated Rab10 mutant RAW264.7 cells were stimulated with LPS for 12 h. **K** EGFR expression on the surface of BMDM was analyzed by flow cytometry (*n* = 3). **L** Percentage of EGFR-positive BMDM is shown (*n* = 3). **M** Mean fluorescence intensity (MFI) is shown (*n* = 3). The graphs depict mean ± SD based on three independent experiments. *P < 0.05, **P < 0.01, ***P < 0.001.

In search of a cellular regulatory mechanisms of cell surface expression of EGFR during endotoxemia described above, we focus on Ras family of small G-proteins. Rab10 is mainly involved in protein trafficking from the Golgi apparatus to the plasma membrane [24]. Here we showed that Rab10 localized with both Golgi and EEA1 but not with late endosomes (Fig. 5A). Meanwhile, we observed a high degree of colocalization between Rab10 and EGFR in RAW264.7 cells by both confocal immunofluorescence and coimmunoprecipitation (Fig. 5B–D). In addition, cell surface EGFR expression was markedly reduced in Rab10 silenced RAW264.7 cells compared with scrambled shRNA-treated cells (Fig. 5E–G).

In order to further clarify the link between Rab10 and EGFR trafficking, it is necessary to validate whether the kinase activity of Rab10 is directly involved in the translocation of EGFR from the Golgi apparatus to cell membrane. We firstly constructed Rab10 plasmids with different mutation sites and established stable cell lines [25]. This is expected, an inactive mutant of Rab10 (Rab10-T23N) localized with the Golgi and distributed in the cytosol, whereas the active mutant (Rab10-Q68L) localized mainly in the early endosomal compartment (Fig. 5H–J). Re-expression of Rab10-WT or Rab10-Q68L up-regulated EGFR surface expression in RAW264.7 cells (Fig. 5K–M). Taken together, these results indicated that Rab10 promotes EGFR trafficking to the cell surface from the Golgi.

### Inhibition of EGFR phosphorylation suppresses glycolysis-dependent M1 polarization in macrophages

**Fig. 6:**
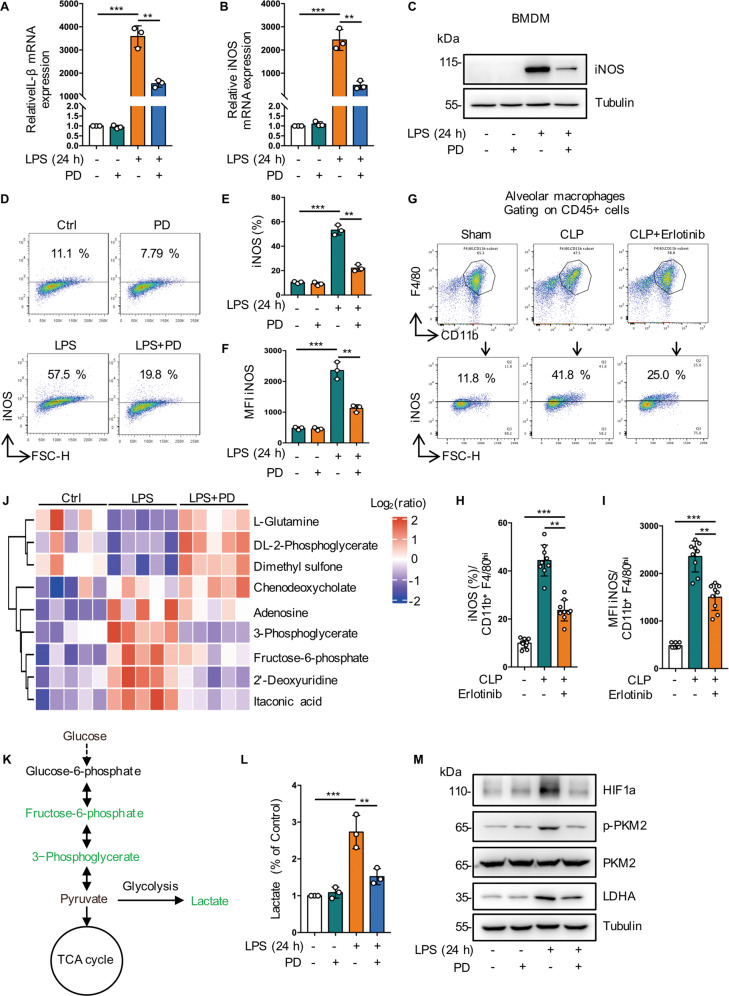
Inhibition of EGFR phosphorylation suppresses glycolysis-dependent M1 polarization in macrophages. **A**–**F** BMDMs were treated with LPS (1 μg/mL) for 24 h with or without PD168393 (10 μM) pretreatment for 30 min. **A** RT-qPCR analysis of mRNA expression of IL-1β (*n* = 3). **B** RT-qPCR analysis of mRNA expression of iNOS (*n* = 3). **C** Western blot was used to detect the expression of iNOS. **D** iNOS expression on the surface of BMDM was analyzed by flow cytometry. **E** Percentage of iNOS -positive BMDM is shown (*n* = 3). **F** Mean fluorescence intensity (MFI) is shown (*n* = 3). **G**–**I** Macrophages were collected from bronchoalveolar lavage fluid of C57BL/6 mice subjected to CLP and were divided into sham-operated, CLP and CLP plus Erlotinib (100 mg/kg, gavage) pretreatmend for 2 h, and alveolar macrophages were identified with CD45 + CD11b + F4/80high. **G** iNOS expression on the surface of alveolar macrophage was analyzed by flow cytometry. **H** Percentage of iNOS-positive alveolar macrophage is shown (*n* = 9). **I** Mean fluorescence intensity (MFI) is shown (*n* = 9). **J** Cluster analysis of differentially expressed metabolites in RAW264.7 measured after treated with LPS (1 μg/mL) for 30 min with or without PD168393 (PD 10 μM) pretreatment for 30 min, when compared with control. Blue and red indicates down-or upregulation, respectively (*n* = 3 samples of each condition). **K** Schematic illustrating the metabolites that are decreased (blue) in PD168393 (10 μM) RAW264.7 cells at 24 h after LPS stimulation. **L** PD168393 (10 μM) pretreatment RAW264.7 cells exhibited a ~2 -fold decrease in lactate levels compared with LPS group (*n* = 3). **M** Representative western blots of HIF1-a, p-PKM2, PKM2, LDHA expression in RAW264.7 cells. The graphs depict mean ± SD based on three independent experiments. *P < 0.05, **P < 0.01, ***P < 0.001.

To evaluate the effect of membrane EGFR activation in M1/M2 phenotypic balance of macrophages in sepsis, we used LPS to stimulate BMDMs and RAW264.7 cells toward M1 phenotype. We found that PD168393 significantly reduced the expression of M1 markers such as iNOS and IL-1β at the RNA level (Figs. 6A, B and S2A). In addition, compared to LPS-treated cells, western blot (Figs. 6C and S2B) and flow cytometry analysis (Figs. 6D–F and S2C–F) showed that the expression level of iNOS was also inhibited in the PD168393 preconditioning cells. In addition, Erlotinib (100 mg/kg, gavage administration) pretreatment effectively downregulated the expression levels of iNOS in bronchoalveolar lavage fluids (BALF) from mice subjected to CLP procedure (Fig. 6G–F). These results indicate that EGFR phosphorylation on cell surface promotes endotoxemia- or sepsis- related M1 macrophage activation.

To further clarify the effect of EGFR on endotoxemia metabolism, we performed a global metabolomic analysis in different groups. We observed that the pentose phosphate pathway (PPP) and glycolysis intermediates were increased in LPS group, while decreased in LPS + PD168393 group, and the decreased of lactate was validated (Fig. 6J–L). Pyruvate Kinase M2 (PKM2) regulates HIF-1ɑ activity and IL-1β induction, and is a critical determinant of the Warburg effect in LPS activated macrophages [26]. We found that PD168393 inhibited PKM2 phosphorylation and reduced HIF-1ɑ and lactate dehydrogenase A (LDHA) protein expression (Fig. 6M). Also, macrophage M1 polarization was inhibited in EGFR knockout BMDM (Figs. S5A–D and S5G–I). These results strongly suggest that inhibition of EGFR phosphorylation can decrease macrophage glycolysis in response to LPS.

### Inhibition of EGFR phosphorylation promote M2 polarization by regulating glutamine metabolism through activation of PPARγ

**Fig. 7:**
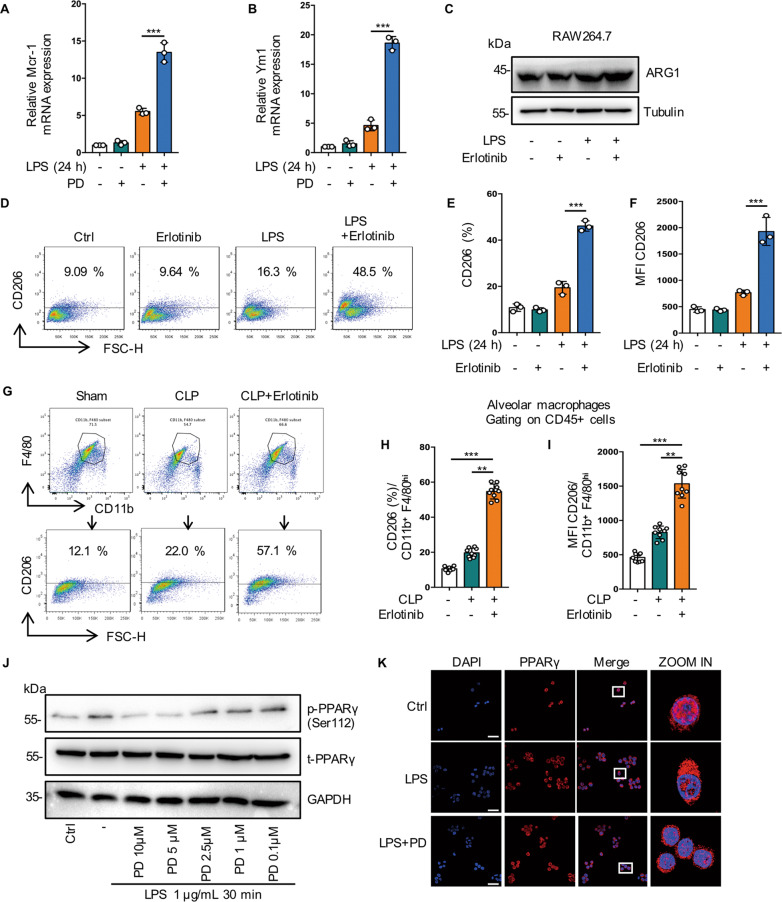

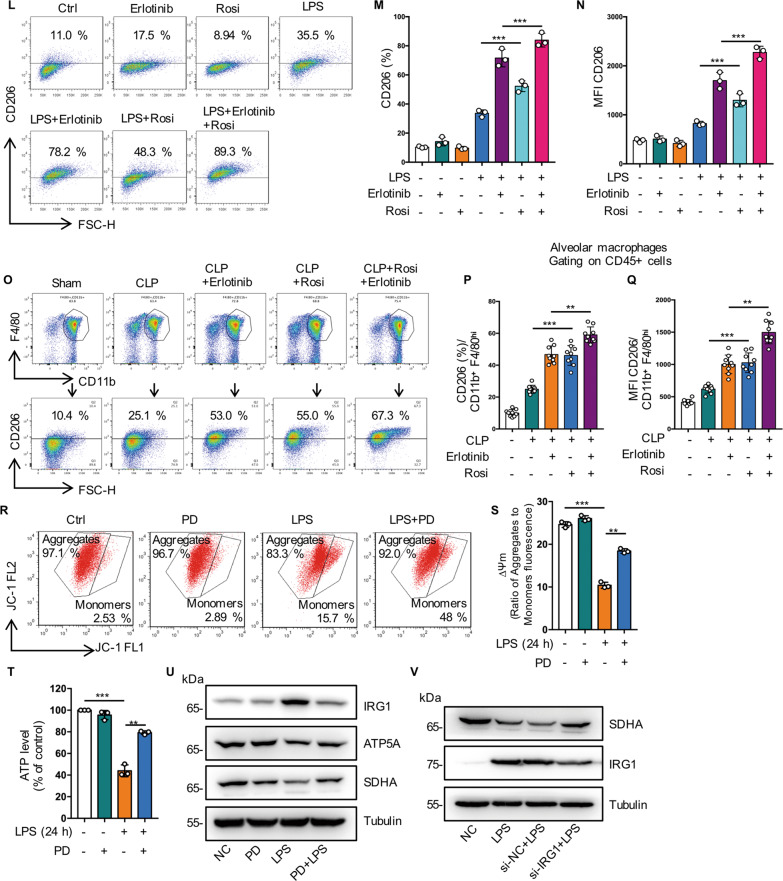
Inhibition of EGFR phosphorylation promote M2 polarization by regulating glutamine metabolism through activation of PPARγ. **A**–**F** RAW264.7 macrophages were treated with LPS (1 μg/mL) for 24 h, with or without Erlotinib (20 μM) pretreatment for 30 min. **A** RT-qPCR analysis of mRNA expression of M2-related genes Mcr1 (*n* = 3). **B** RT-qPCR analysis of mRNA expression of M2-related genes Ym1 (*n* = 3). **C** Representative western blot of Arg1. **D** Flow cytometry analysis showing the level of M2 macrophage-associated markers CD206. **E** Percentage of CD206-positive RAW264.7 is shown (*n* = 3). **I** Mean fluorescence intensity (MFI) is shown (*n* = 3). **G**–**I** Macrophages were collected from bronchoalveolar lavage fluid of C57BL/6 mice subjected to CLP and were divided into sham-operated, CLP and CLP plus Erlotinib (100 mg/kg, gavage) pretreatmend for 2 h, and alveolar macrophages were identified with CD45 + CD11b + F4/80high. **G** CD206 expression on the surface of alveolar macrophage was analyzed by flow cytometry. **H** Percentage of CD206-positive alveolar macrophage is shown (*n* = 9). **I** Mean fluorescence intensity (MFI) is shown (*n* = 9). **J** Immunoblot analysis of p-PPARγ (Ser112), t-PPARγ in RAW264.7 cells treated with LPS (1 μg/mL) for 30 min with or without indicated concentration of PD168393 (10 μM) pretreatment for 30 min. **K** Fluorescence images depicting PPARγ translocation (left panel, scale bar, 50 μm; right panel, scale bar, 5 μm). **L**–**N** RAW264.7 cells were treated with LPS (1 μg/mL) for 24 h with or without Erlotinib (10 μM) or Rosiglitazone (Rosi (20 μM) pretreatment. **L** Cell surface CD206 were analyzed by flow cytometry. **M** Percentage of CD206-positive RAW264.7 is shown (*n* = 3). **N** Mean fluorescence intensity (MFI) is shown (*n* = 3). **O**–**Q** Macrophages were collected from bronchoalveolar lavage fluid of C57BL/6 mice subjected to CLP and were divided into Sham-operated, CLP and CLP plus Erlotinib (100 mg/kg, gavage) pretreatmend for 2 h, and alveolar macrophages were identified with CD45 + CD11b + F4/80high. **O** CD206 expression on the surface of alveolar macrophage was analyzed by flow cytometry. **P** Percentage of CD206-positive alveolar macrophage is shown (*n* = 9). **Q** Mean fluorescence intensity (MFI) is shown (*n* = 9). **R**–**V** RAW264.7 cells were treated with LPS (1 μg/mL) for 30 min with or without PD168393 (10 μM) pretreatment for 30 min. **R** Flow cytometry analysis of JC-1 for the detecting the change of mitochondrial membrane potential (ΔΨm) (left panel, JC-1 aggregates; right panel, JC-1 monomers). **S** The ratio of JC-1 aggregates /JC-1 monomers was calculated as Δψm. **T** Total cellular ATP level was detected (*n* = 3). **U** Immunoblot analysis of IRG1, ATP5A, SDHA, Tubulin as a loading control. **V** Immunoblot analysis of SDHA and IRG1 in Control or IRG1 silenced RAW264.7 cells, Tubulin as a loading control. The graphs depict mean ± SD based on three independent experiments. *P < 0.05, **P < 0.01, ***P < 0.001.

Previous reports show that M2 macrophages was induced by metabolic disorders, which promoted M2 polarization and inhibited metaflammation [27, 28]. Next, we investigated the role of EGFR phosphorylation in M2 phenotype polarization. We found that Erlotinib upregulated the expression of M2 markers including Mrc1 at the RNA level (Figs. 7A, B and S3A). Compared with LPS-treated cells, western blot showed increased Arg1 expression (Figs. 7C and S3B) and flow cytometry analysis showed the expression level of CD206 increased more than 2-fold in Erlotinib pretreatment cells (Figs. 7D–F and S3C, D). Furthermore, Erlotinib (100 mg/kg, gavage administration) pretreatment effectively upregulated the expression level of CD206 in BALF macrophages from mice subjected to CLP procedure (Fig. 7I, J). All these results indicate that inhibition of EGFR phosphorylation promotes M2 macrophage polarization during sepsis.

PPARγ controls macrophage glutamine metabolism, providing a link between transcription, M2 polarization, and metabolism and glutamine is required for M2 polarization [29–31]. Our metabonomics analysis indicated that glutamine levels increased in PD168393 (PD) treated group (Fig. 6J). We found that PD suppressed the phosphorylation of PPARγ in a concentration-dependent manner (Fig. 7J). In addition, compared with the LPS treated group, we observed that PD markedly promoted the activation of PPARγ, indicating increased PPARγ nuclear transfer in MH-S cells (Fig. 7K). RT-PCR results showed that the expression of Arg1, MRC1 and YM1 were increased after PPARγ activation induced by ROSI (PPARγ activator) (Fig. S3E–G). Flow cytometry indicated that the expression of CD206 increased in accordance with the activation of PPARγ in vitro (Fig. 7L–N) and in vivo (Fig. 7O–Q).

Glutamine metabolism is important for the TCA cycle turnover and mitochondrial oxidative phosphorylation [32]. In LPS-induced endotoxemia, Erlotinib pretreatment could maintain mitochondrial membrane potential (ΔΨm) levels and ATP content, suggesting that EGFR is involved in energy metabolism during macrophage activation (Fig. 7R–T). PPARγ depletion from macrophages upregulates immunoresponsive gene 1 (IRG1) expression, which subsequently led to itaconic acid accumulates and blocks Mitochondrial succinate dehydrogenase (SDH) activity [29]. Mutation in the A subunit of SDH (SDHA) diminishes enzymatic activity and thereby impair OXPHOS [33]. Metabolomics data showed that itaconic acid level increased in the LPS group while decreased significantly in the PD168393 + LPS group (Fig. 6J). Meanwhile, we found that PD168393 reduced IRG1 protein expression levels and upregulated the expression of mitochondrial respiratory chain subunits ATP5A (complex V) and SDHA (Complex II) compared with LPS-induced RAW264.7 cells (Fig. 7U). Furthermore, IRG1 knock-down restored the expression of SDHA (Fig. 7V). These results suggest that EGFR is involved in maintaining OXPHOS activity and inhibition of EGFR phosphorylation promotes M2 polarization by regulating glutamine metabolism through activation of PPARγ.

### Inhibition of EGFR phosphorylation switches M1 phenotype to M2 phenotype and alleviates acute lung injury induced by sepsis

**Fig. 8:**
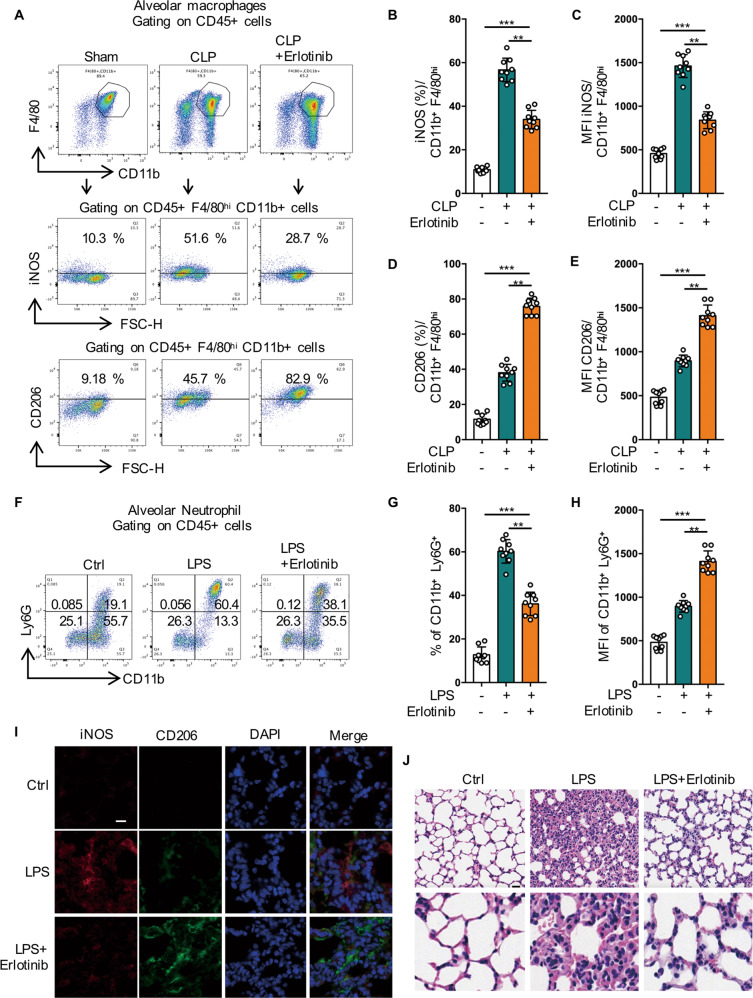
Inhibition of EGFR phosphorylation switches M1 phenotype to M2 phenotype and alleviates acute lung injury induced by sepsis. **A**–**E** Macrophages were collected from bronchoalveolar lavage fluid of C57BL/6 mice subjected to CLP and were divided into sham-operated, CLP and CLP plus Erlotinib (100 mg/kg, gavage) pretreatmend for 2 h, and alveolar macrophages were identified with CD45 + CD11b + F4/80high. **A** iNOS and CD206 expression on the surface of alveolar macrophage were analyzed by flow cytometry. **B** Percentage of iNOS-positive alveolar macrophage is shown (*n* = 9). **C** Mean fluorescence intensity (MFI) of iNOS is shown (*n* = 9). **D** Percentage of CD206-positive alveolar macrophage is shown (*n* = 9). **E** Mean fluorescence intensity (MFI) of CD206 is shown (*n* = 9). **F**–**J** Mice were injected intraperitoneally with LPS (20 mg/kg) or an equal volume of PBS, after pretreatment with Erlotinib (100 mg/kg) for 2 h. **F** The BALF neutrophils were analyzed by flow cytometry with neutrophils markers (CD11b and Ly6G). **G** Percentage of CD11b + Ly6G+ positive alveolar macrophage is shown (*n* = 9). **H** Mean fluorescence intensity (MFI) of CD11b + Ly6G+ is shown (*n* = 9). **I** Immune-staining of iNOS and CD206 in Lung sections. **J** Lung sections were stained with hematoxylin and eosin (H&E). Scale bar, 50 μm (upper panel) and 5 μm (lower panel). The graphs depict mean ± SD based on three independent experiments. *P < 0.05, **P < 0.01, ***P < 0.001.

To further validate the effects of EGFR inhibitor on inflammation and macrophage polarization in vivo, both LPS- and CLP-induced acute lung injury (ALI) murine model were applied [34]. We found that in Erlotinib (100 mg/kg) administered mice, the expression of M2 marker CD206 increased, while the expression of M1 marker iNOS decreased in macrophages derived from mouse alveolar lavage fluid (Fig. 8A–E). We also observed that Erlotinib administered enhanced the expression of CD206 and inhibited the expression of iNOS with immunofluorescence (Fig. 8I). In addition, Erlotinib administered obviously reduced the BALF neutrophil infiltration in the lungs of LPS- induced lung injury (Fig. 8F–H). Furthermore, H&E staining revealed that LPS (20 mg/kg) injection led to inflammatory cells infiltration, interstitial edema, and interalveolar septal thickening at 24 h after treatment, while Erlotinib treatment attenuated the pathological changes in the lung tissues (Fig. 8J). Collectively, these results indicate that EGFR inhibitor may ameliorate septic ALI through regulating macrophage polarization and reducing inflammation in vivo.

## Discussion

Our previous study showed that cell surface expression of the TLR4 is increased in BMDMs during LPS-induced endotoxemia [13]. Interestingly, we found that the expression of cell surface EGFR was also increased in LPS-induced inflammation in macrophages, including mouse macrophage cell lines, mouse primary macrophages, and clinical sepsis patient primary monocytes. However, EGFR inhibitor (PD168393/Erlotinib) inhibited LPS-mediated increased EGFR expression on the macrophage cell surface. This may explain, at least partially, why LPS can also increase EGFR-mediated effects such as cell proliferation [35, 36]. Cell surface expression of receptors is determined by the balance between receptor trafficking from the Golgi apparatus to the cell membrane and internalization into endosomal compartments from the membrane [37, 38].

The small Rab GTPase, Rab10, is mainly involved in protein trafficking from the Golgi apparatus to the plasma membrane [24]. Previous studies showed continuous replenishment of TLR4 from Golgi to plasma membrane is regulated by Rab10 [39]. We found that Rab10 active-site mutant altered the expression of cell surface EGFR in RAW264.7 macrophages during endotoxemia but did not affect total protein levels of EGFR expression. Meanwhile, knockdown of Rab10 decreased EGFR expression on RAW264.7 cells surface. Furthermore, Rab10 was highly colocalized with EGFR in macrophages. These results suggest that Rab10 influences EGFR surface expression by promoting its translocation.

Endocytosis of receptors has been considered as a mechanism of signal attenuation via receptor and ligand clearance from the cell surface [40, 41]. Rab5a is a marker of early sorting endosomes [42, 43]. Our study showed that Rab5a mediated the early internalization of EGFR in macrophages. When we inhibited the phosphorylation of EGFR, the activation of Rab5a and the co-localization between EGFR and Rab5a was inhibited, which led to the dysfunction of EGFR endocytosis. As our results showed, PD168393 reduced the co-localization of EGFR and Rab5a and inhibited the activation of Rab5a, which led to the blockade of the early endocytic transport of EGFR was blocked, thus preventing the decrease of EGFR expression on the surface of BMDM after 1 h treatment with LPS. The internalization and lysosomal-mediated degradation of EGFR is an important negative feedback mechanism to regulate the amplitude and kinetics of EGFR signaling [44, 45].

Rab7a regulates the early to late endosome transport and is a key regulator of the late endocytic pathway [46, 47]. We showed that phosphorylation of Rab7a blocked the late EGFR endocytosis and inhibited the lysosomal degradation pathway of EGFR, which led to ultimately promoting the cell membrane transport of EGFR and increasing the level of EGFR on the cell membrane. We found that LPS induced Rab7a phosphorylation at S72, while PD168393 inhibited this phosphorylation, which inactivated Rab7a and resulted in the destruction of Rab7a-mediated late EGFR endocytosis. When Rab7a is phosphorylated, the co-localization of CD63 with Rab7a and the co-localization of EGFR with CD63 and LAMP1 are decreased. As lysosomal degradation is the terminal stages of membrane receptors endocytosis, our results converge with recent findings to suggest that phosphorylation of Rab7a impedes the late endocytic transport of EGFR [48–50]. In addition, phosphorylation of Rab7a promotes LPS-induced EGFR membrane expression, activated macrophages, and promotes lung tissue injury in mice with endotoxemia. MAPK signaling activated by Rab7a phosphorylation may be a response to post-infectious inflammation, and Rab7a may be a new target for the control of LPS/EGFR-mediated inflammation and autoimmune diseases.

Differences in the bioenergetic demands of M1 and M2 macrophages are emerging as regulatory circuits that adjust macrophage behavior in response to nutrient states in its habitation and the infected tissues [51]. M1 macrophages rely on aerobic glycolysis to produce ATP, increasing glucose and glutamine consumption, but they inhibit oxidative metabolism [52, 53]. In contrast, M2 macrophages maintain a complete TCA cycle and promote oxidative metabolism [54, 55]. In the present study, we found that Erlotinib not only inhibited glycolysis to down-regulate LPS-induced M1 polarization, but also promoted M2 polarization through the PPARγ pathway. We describe a novel approach by which EGFR is involved in endotoxemia by regulating the M1/M2 phenotype transformation of macrophages through cell metabolism.

In conclusion, we demonstrated for the first time that cell surface expression of EGFR is enhanced in the macrophage in response to LPS. Then we elaborated on the entire cell transport process of EGFR, including plasma membrane translocation, early internalization and late endocytosis, and the specific Rab proteins that regulate these processes, respectively. Moreover, we found that cell surface EGFR level regulates the M1/M2 polarizing phenotypic transformation of macrophages and influences sepsis-induced multiple organ injury through metabolic reprogramming.

## Materials and methods

### Antibodies

Antibodies against α-Rab5a (#2143), α-Rab7a (#9367), α-Rab10 (#8127), α-EGFR (#4267), α-Phospho-EGF Receptor (Tyr1068) (#2234 S), α-Phospho-p38 MAPK (Thr180/Tyr182) (#2775 S), α-p38 MAPK (D13E1) XP Rabbit mAb (#8690), α-p44/42 MAPK (Erk1/2) (#695), α-IRG1 (#17805) were purchased from Cell Signaling Technology. Antibodies against α-HIF1-α (sc-53546), α-EGFR (sc-120), α-PKM2 (sc-365684), α-LDHA (sc-133123), α-MAPK14 (sc-11415), α-EGFR (sc-120), α-EEA1 (sc-137130), α-TGN38 (sc-166594), α-LAMP1 (sc-20011), α-PPARγ (sc-7273), α-SDHA (sc-390381), α-COX2 (sc-514489), α-COX4 (sc-517553), α-ATP5A (sc-136178) were purchased from Santa Cruz Biotechnology. Antibodies against α-HA (51064-2-AP, proteintech Group), α-FLAG (20543-1-AP, proteintech Group), APC anti-mouse CD11b (APC-65055, proteintech Group) were purchased from proteintech Group. Antibodies against APC anti-mouse iNOS (#85-17-5920-82, eBioscience), PE anti-mouse CD206 (MMR) Antibody (#85-12-2061-82, eBioscience), α-F4/80 (11-4801-82, FITC, eBioscience), PE anti-mouse Ly6G (#12-9668-82, eBioscience), α-CD14 (25-0149-42, FITC, eBioscience) were purchased from eBioscience. Antibodies against α-GAPDH (T0004), α-β-Tubulin (T0023), α-Phospho-PPAR gamma (Ser112) (AF3284), α-Phospho-PKM2 (AF7231) were purchased from Affinity Biosciences.

### Animal

C57BL/6 (WT) mice were obtained from Jiangsu Jicui Yaokang Biotechnology Co., Ltd. (Jiangsu, China). Rab5a knockout mice, EGFR^flox/flox^ mice and Lyz2-Cre mice were obtained from Cyagen Biosciences (Guangzhou, China). Myeloid-specific EGFR conditional knock out (CKO) mice (EGFR-CKO mice) was generated previously in our laboratory, in which EGFR is specifically deleted in myeloid macrophages by crossing EGFR^flox/flox^ mice with Lyz2-Cre mice (EGFR^f/f^; Lyz2-Cre). Experiments were performed with male mice (6–8 weeks old). The following treatments were administered: LPS (20 mg/kg, i.p.); Erlotinib (100 mg/kg dissolved in Captisol, gavage); Rosiglitazone (Rosi) (30 mg/kg, gavage). All animal experiments were reviewed and approved by the Animal Ethics Committee of Guangdong Medical University.

### Cecal ligation and puncture

CLP was performed as described previously [56]. Briefly, 6–8-week-old C57Bl/6 mice were anesthetized and kept warm using a heating pad. An abdominal midline incision was made, and the caecum was ligated at about a quarter of the distance from the luminal entry to its tip. The cecum was perforated once through and through in the midsection using a 20-gauge needle. A small amount of the caecal content was gently pushed out of the four openings into the peritoneum. Subsequently, the abdominal muscles were sutured and the skin was closed with two staples. The intestine was returned to the peritoneal cavity. 1 mL of saline was injected subcutaneously to the mice after the incision was closed. In sham-operated animals, the cecum was isolated, but neither ligated nor punctured.

### Bone marrow derived macrophage (BMDM) isolation and cells culture

BMDM were isolated from the femurs and tibias of C57BL/6 mice, as previously described [51]. Murine-derived macrophage RAW264.7 cells (C7505) and MH-S cells were purchased from the (Beyotime Biotechnology, Shanghai, China). Cells were cultured in 10% FBS DMEM and containing 1% (v/v) penicillin/streptomycin.

### Preparation of peritoneal macrophages

Peritoneal macrophages were harvested 24 h after LPS (10 mg/kg) intraperitoneal injection with or without Erlotinib (100 mg/kg, Intragastric administration). Briefly, PBS or normal saline was injected into the abdominal cavity and extracted after gentle shaking. The peritoneal lavage suspension was centrifuged at 400 g. After centrifugation, cells were resuspended in cold 1% BSA-PBS and used immediately for Flow cytometry analysis.

### Preparation of human peripheral blood mononuclear cells (PBMCs)

PBMCs were isolated from whole blood of healthy human volunteers or ICU patients meeting sepsis criteria. Blood was collected in 10 mL EDTA blood tubes, and immediately layered onto Ficoll medium and stratified via gradient centrifugation. The PBMC layer was isolated and used immediately for Flow cytometry analysis. The collection of PBMCs complied with all relevant ethical regulations and was approved by the Human Ethics Committee of Guangdong Medical University.

### Flow cytometry analysis

Macrophages or PBMCs collected from mouse or human donors were incubated with F4/80 or CD14 antibody for 30 min on ice. To measure membrane of EGFR, macrophages were stained with PE-EGFR antibody for 30 min on ice. The fluorescence signal was analyzed on at least 10,000 F4/80 positive events by FACScalibur cytometer (BD Biosciences, USA), and data processing was performed using FlowJo 10.0 version software.

### RNA extraction and Real-time PCR

Total RNA was prepared from BMDM, MH-S or RAW264.7 cells. Real-time PCR was performed with a Light Cycler 480 (Roche) real-time PCR system and analyzed by Bio-Rad iQ5 software. The gene-specific primers were listed as below: Rab5a (forward: GCTAATCGAGGAGCAACAAGAC; reverse: CCAGGCTTGATTTGCCAACAG), EGFR (forward: GCCATCTGGGCCAAAGATACC; reverse: GTCTTCGCATGAATAGGCCAAT), IL-1β (forward: GCAACTGTTCCTGAACTCAACT; reverse: ATCTTTTGGGGTCCGTCAACT), iNOS (forward: GAGACAGGGAAGTCTGAAGCAC; reverse: CCAGCAGTAGTTGCTCCTCTTC), Arg1 (forward: CATTGGCTTGCGAGACGTAGAC; reverse: GCTGAAGGTCTCTTCCATCACC), Ym1 (forward: TACTCACTTCCACAGGAGCAGG; reverse: CTCCAGTGTAGCCATCCTTAGG), Mcr-1 (forward: GTGAGTCTGGTGGAGAATGTGC; reverse: GTAGTCTCCAGCACGATGCTGA), GAPDH (forward: CATCACTGCCACCCAGAAGACTG; reverse: ATGCCAGTGAGCTTCCCGTTCAG).

### siRNA knockdown

RAW264.7 or BMDM cells (4 × 10^5^ cells) were seeded 6-well plate. 200 pmol per well of indicated specific siRNA or non-specific siRNA was transfected using Lipo-RNAiMAX Reagent (Invitrogen) following the manufacturer’s instructions. After 48 h transfection, Western blot was used to validate the efficiency of the corresponding gene knockdown. The siRNAs used in these studies were synthesized by GenePharma (Shanghai, China): non-specific (ns) siRNA (5′–CUACGUCCAGGAGCGCACC–3′), Rab5a (5′–ACAGUUUGAGGUACUGUUC–3′), Rab10 (5′–AACGATTTCACACCATCACAA–3′), IRG1 (5′–GAGGAUGAUUCUAGACACUTT–3′).

### shRNA knockdown

Plasmids of shRNA targeting Rab10, Rab5a, Rab7a and MAPK14 and negative control shRNA were designed and synthesized by GenePharma (Shanghai, China). The indicated shRNA Lentiviral virus were packed in 293 T cells. Cells were infected with lentivirus and selected using 2 μg/mL puromycin for 72 h. The shRNAs used in these studies were purchased from GenePharma (Shanghai, China). Target sequences: Rab10 shRNA, AACTGGAACAGACAAACTATC; Rab7a shRNA, AAGACCAGACGCCACTCCAAC; MAPK14 shRNA, CTCAGAGTCTGCAAGAAACTA.

### Immunoprecipitation and immunoblot

Cells were suspended in IP lysis buffer (Thermo, CA, USA) for 30 min on ice with continuous mixing. After centrifugation at 12,000 × *g* for 15 min, a final volume of 500 μl supernatants (500 μg protein) was precleared for 1 h with 20 μl of a 50% slurry of Protein A/G magnetic bead (Merck Millipore, CA, USA) at 4 °C. We used the magnetic stand to capture the beads and incubated the supernatants with 4 μg of antibodies against mouse EGFR (Santa Cruz Biotechnologies) at 4 °C overnight. 50 μl of Protein-A/G magnetic beads were incubated with the protein solution in a rotary shaker for 2 h at 4 °C. Thereafter, the beads were collected and washed 6 times with 1 mL PBS, the eluents were subjected to SDS-PAGE (6–12% gels) and then transferred to Immobilon-P membranes for Western blotting.

### Immunofluorescence

For cells, RAW264.7 or BMDM cells were seeded in 35 mm Glass Bottom Dish and then subjected to fixation with 4% paraformaldehyde, and permeabilization with 0.1% TritonX-100, then incubated with the indicated primary antibody at 4 °C overnight, after blocked with 5% BSA for 30 min at RT. After incubated with fluorescent conjugated secondary antibodies and DAPI, and then fluorescence signal was observed with an Olympus fluorescent microscope (FV10i-DOC, Olympus, Japan).

### Fluorescence microscope

For histological studies, tissues were fixed, sectioned, and stained as described. Briefly, tissues sections were incubated with iNOS antibody (1: 50; Santa Cruz), and CD206 antibody (1: 100; Proteintech Group) overnight at 4 °C. Subsequently, fluorescence conjugated secondary antibodies were used at TR for 30 min. A confocal microscope (Leica TCS SP8, West Hollywood, CA) was used for imaging samples.

### Western blotting and Phostag-PAGE

For SDS-PAGE, indicated cells were lysed in lysis buffer as above. Lysates (20 μg) (or Appropriate amounts of proteins from anti-Rab7a CO-IP lysates) was then loaded onto the SDS-PAGE and subjected to electrophoresis. For Phostag SDS-PAGE, 100 mM Phostag acrylamide (#F4002, ApexBio Technology) and 60 mM MnCl2 were added to the 12% SDS-PAGE gel. The protein eluents were subjected to electrophoresis on 12% polyacrylamide gel as above. After electrophoresis, gels were transferred onto nitrocellulose membranes and sent to immunoblotting with Rab7a antibody.

### Phosphoproteomics

Phosphoproteomics outcomes were generated by Shanghai Applied Protein Technonlogy Co., Ltd. (Shanghai, China). RAW264.7 cells were prepared from standard cultures following treatment with LPS (1 mg/mL) for 24 h, with or without PD168393 (10 μM) pretreatment for 30 min. Protein lysates were digested into peptides according to published guidelines. Phospho-peptides were enriched and sent to LC–MS/MS analysis, and the data were collected. The differentially expressed phosphorylated peptides were screened according to the criteria of expression fold change of more than 2-fold (up-regulation of more than 2-fold or down-regulation of less than 0.5-fold) and *P* Value < 0.05. The differentially expressed phosphorylated peptides with two or more null values in one group of samples and all null values in the other group were excluded from subsequent bioinformatics analysis. Proteomics experiments are representative of 3 biological replicates performed in 2 technical duplicates.

### Metabolomics

The metabolomic assay and data analysis was performed by Shanghai Applied Protein Technonlogy Co., Ltd. (Shanghai, China). RAW264.7 cells were prepared from standard cultures following treatment with LPS (1 mg/mL) for 24 h, with or without PD168393 (10 μM) pretreatment for 30 min. Protocol for metabolite quantification, data normalization, and quality control methods are given in the published guidelines. Metabolomics data was filtered (relative standard deviation) and normalized. Statistical significance of metabolites was calculated using Student’s *t*-test. According to the Variable Importance for the Projection (VIP) obtained by OPLS-DA model, the influence strength and interpretation ability of the expression patterns of metabolites on the classification and discrimination of each group of samples were measured, and the differential metabolites with biological significance were mined. The differences among the groups were screened preliminarily with VIP > 1 as standard. Further univariate statistical analysis was used to verify the significance of the different metabolites. Metabolites with VIP > 1 and *P* value < 0.05 in multivariate statistical analysis were selected as metabolites with significant differences. Metabolomics experiments are representative of 5 biological replicates performed in 2 technical duplicates.

### Mitochondrial membrane potential assay

The assay was performed using JC-1 Mitochondrial membrane potential assay kit in 6-well format (#C2006, Beyotime) according to manufacturer recommendations. RAW264.7 cells were treated with LPS 24 h, with or without Erlotinib pre-treatment, the assay was performed according to the manufacturer’s instructions. 5 × 10^4^ cells per 1 well of a 6-well plate were used as one sample, and then measured by flow cytometry.

### Measurement of lactic acid concentration

RAW264.7 cells were seeded at 80 to 90% confluence in 6-well plates. Cells were treated with LPS (1 μg/mL) 24 h, with or without PD168393 (10 μM) pretreatment for 30 min, and the content of lactic acid in the medium supernatant was measured. Lactic acid concentration was assessed using Lactic Acid Detection Kit (SenBeiJia, Nanjing, China) according to the protocols and the lactate levels were normalized to protein concentration.

### Reporting summary

Further information on research design is available in the Nature Research Reporting Summary linked to this article.

## Supplementary information

Figure S1

Figure S2

Figure S3

Figure S4

Figure S5

Figure S6

Supplementary Figure legends

Full uncut gels

Supplementary Table 1

Supplementary Table 2

Supplementary Table 3

Supplementary Table 4

Supplementary Table 5

Supplementary Table 6

Study approval

ClinicalTrials identifier

Reporting Summary

## Data Availability

All data are available in the main text or the supplementary materials.
