# Supplementary figures and images for: EGFR tyrosine kinase activity and Rab GTPases coordinate EGFR trafficking to regulate macrophage activation in sepsis
Source: Cell Death Dis. 2022 Nov 7;13(11):934. doi: 10.1038/s41419-022-05370-y (PMC9640671; doi:10.1038/s41419-022-05370-y)

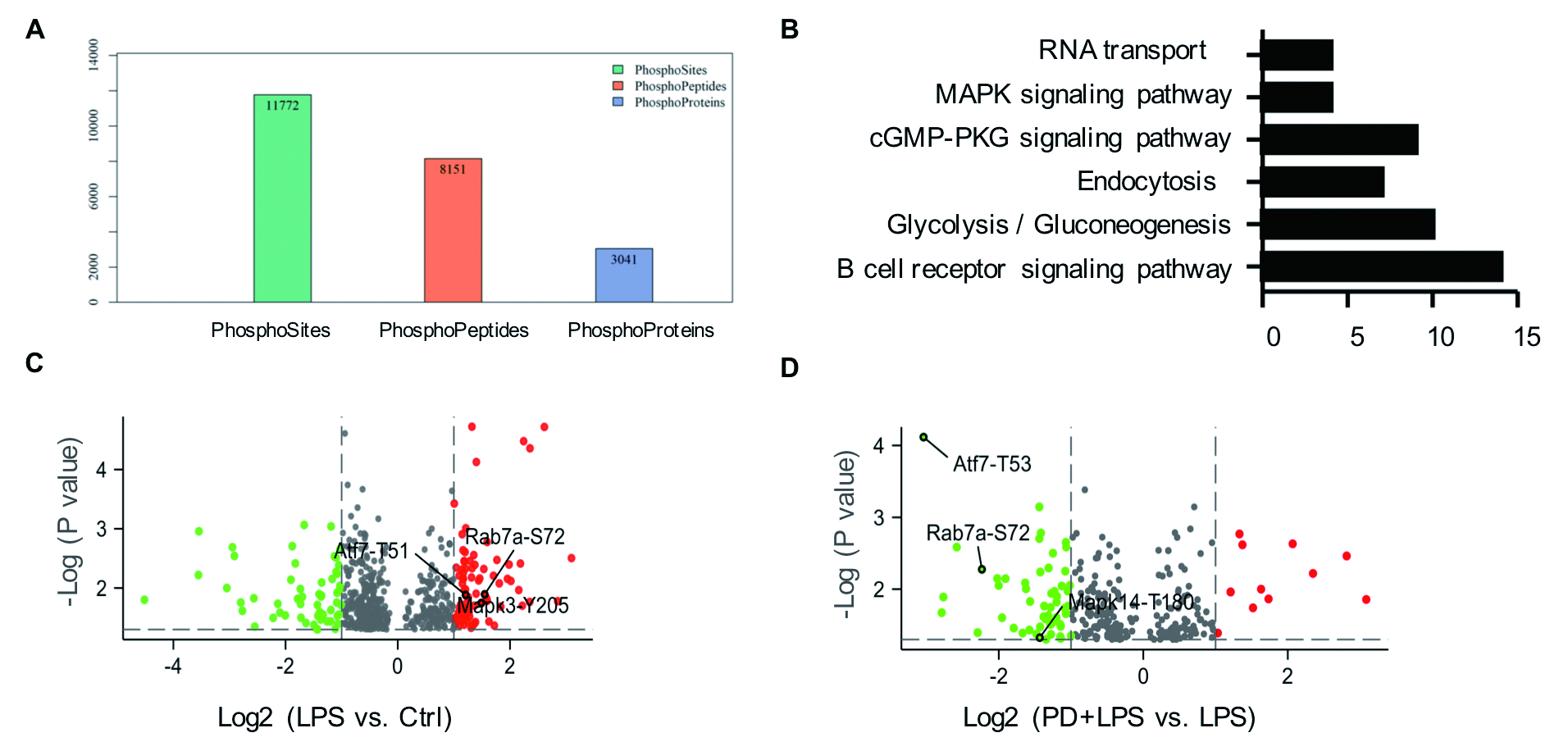

Supplement: Supplementary file 1 — Figure S1 [file 41419_2022_5370_MOESM1_ESM.tif]

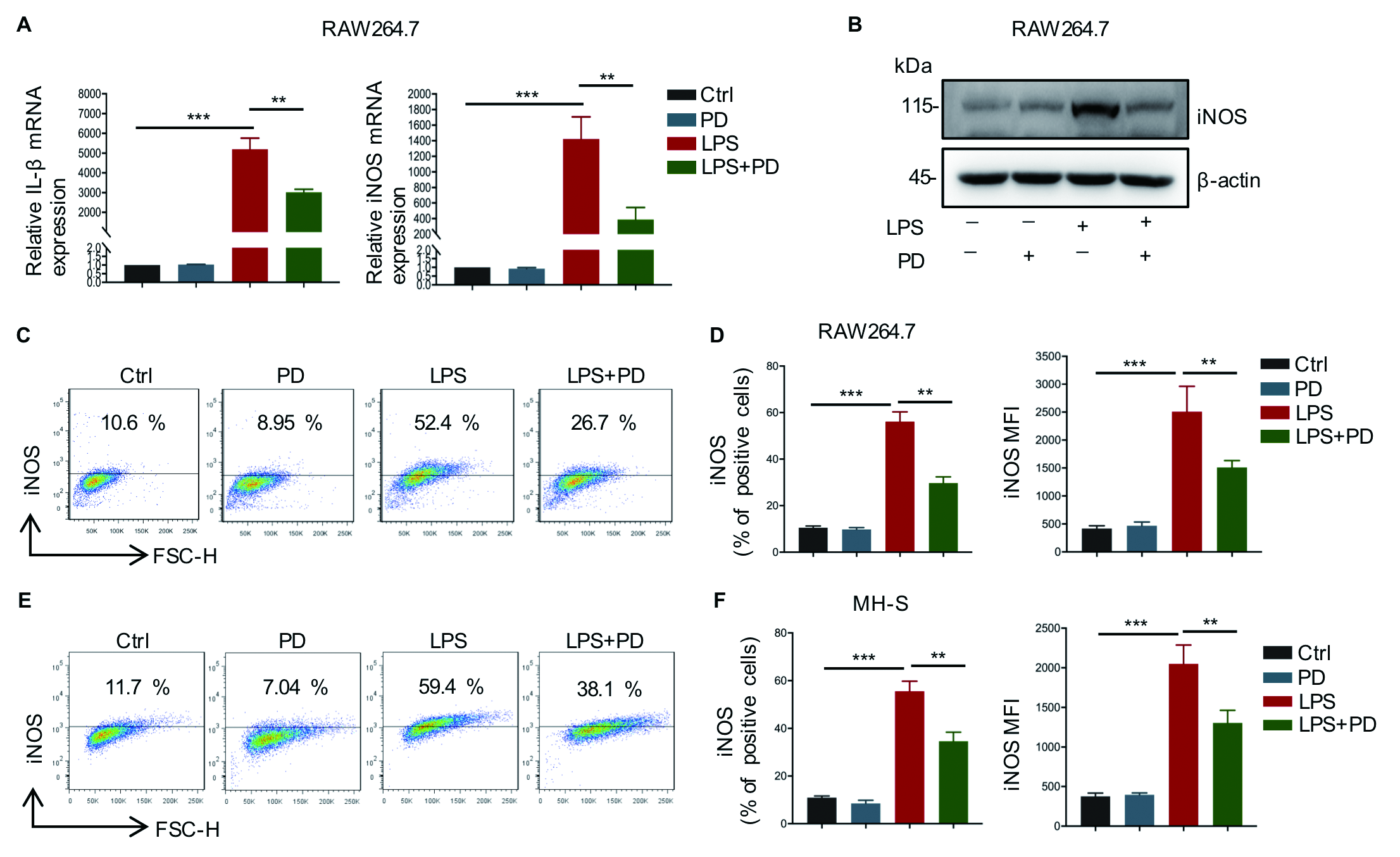

Supplement: Supplementary file 2 — Figure S2 [file 41419_2022_5370_MOESM2_ESM.tif]

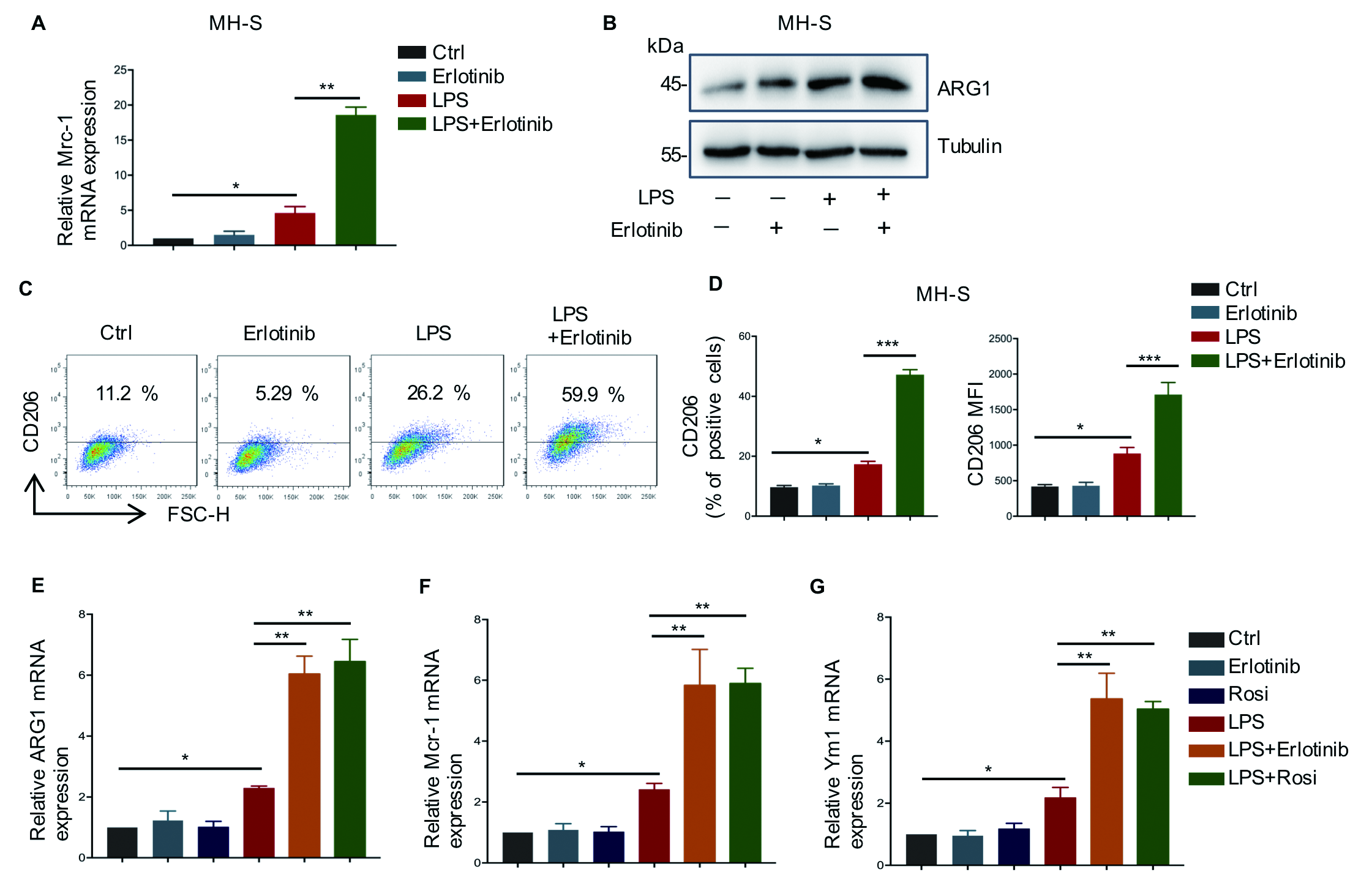

Supplement: Supplementary file 3 — Figure S3 [file 41419_2022_5370_MOESM3_ESM.tif]

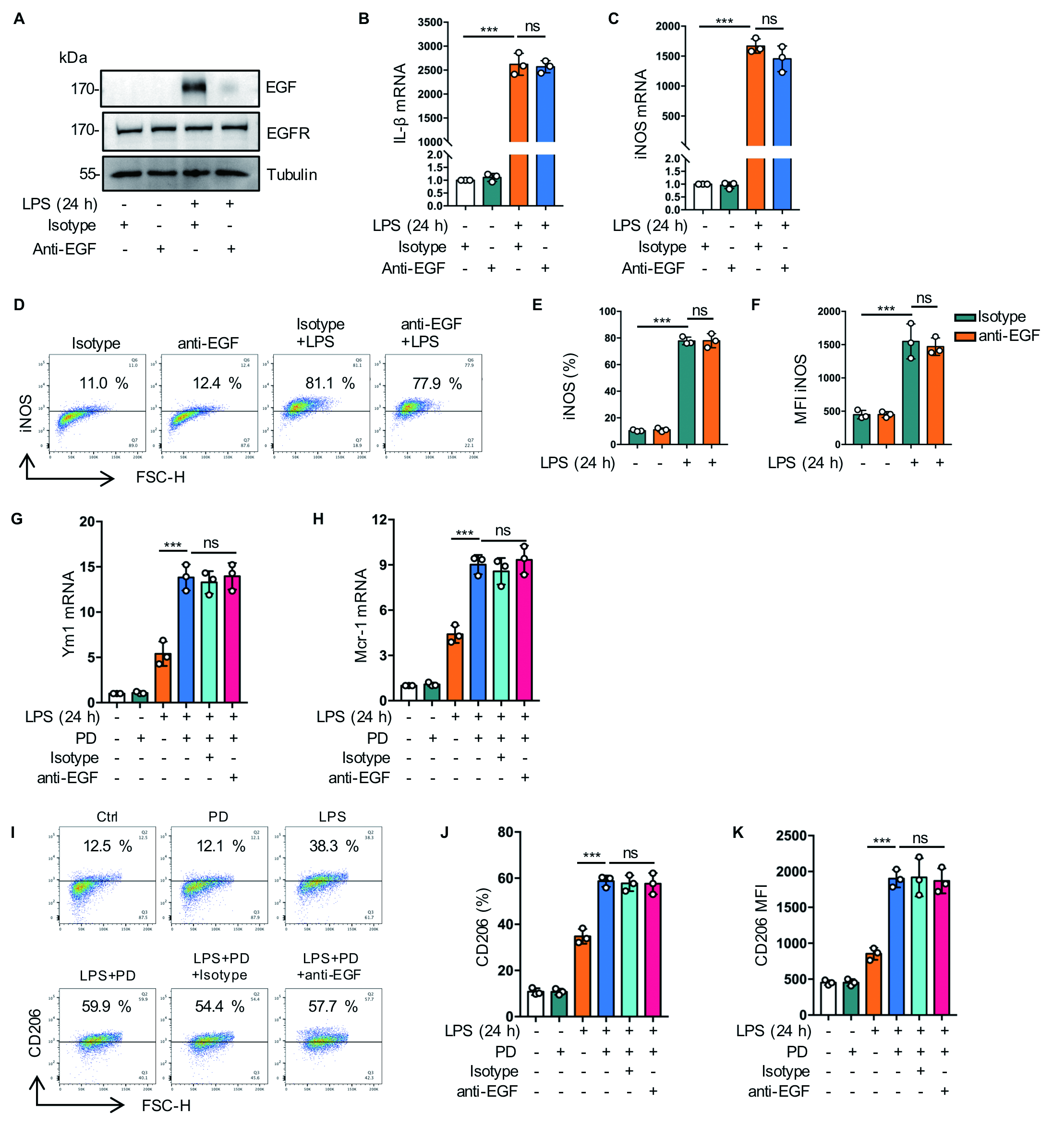

Supplement: Supplementary file 4 — Figure S4 [file 41419_2022_5370_MOESM4_ESM.tif]

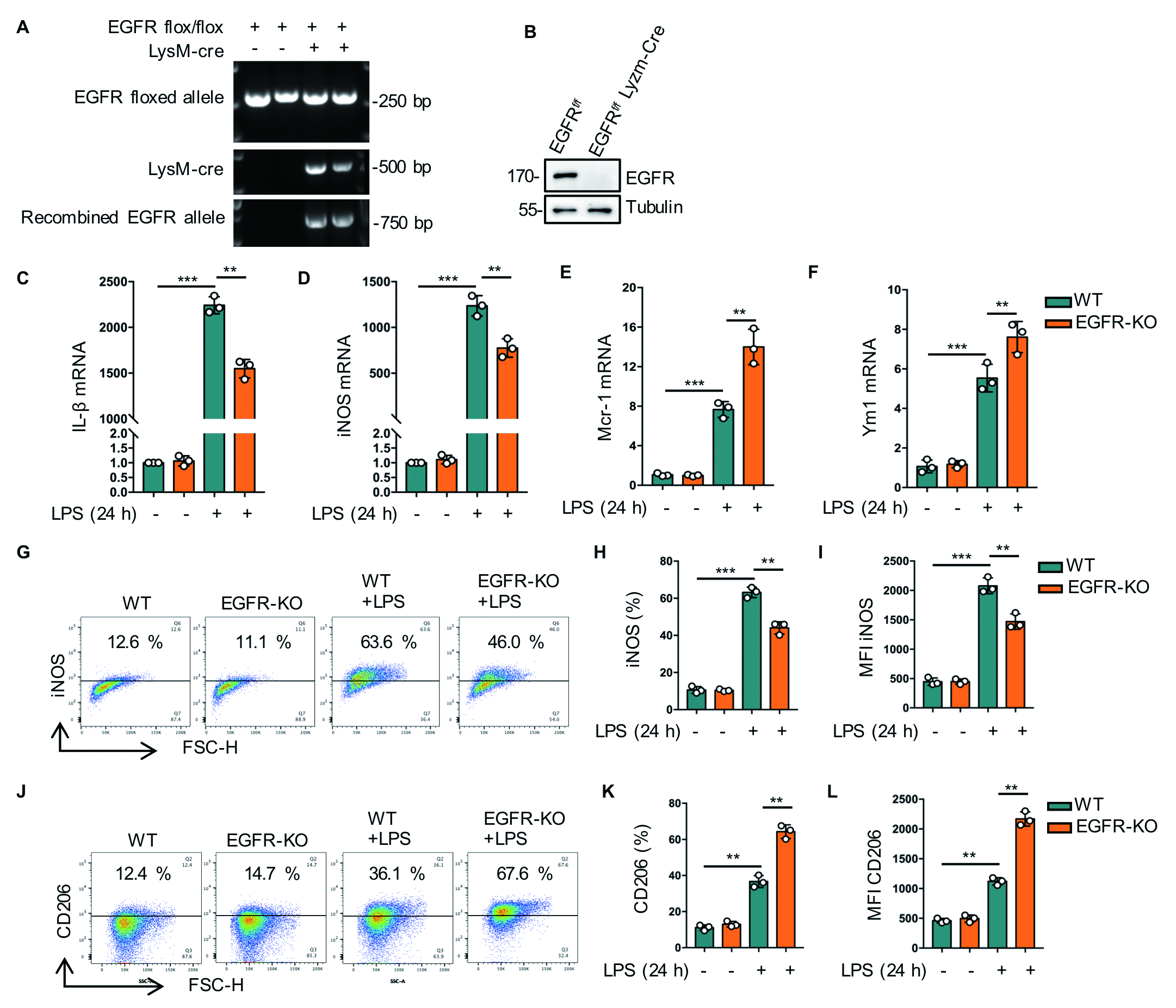

Supplement: Supplementary file 5 — Figure S5 [file 41419_2022_5370_MOESM5_ESM.tif]

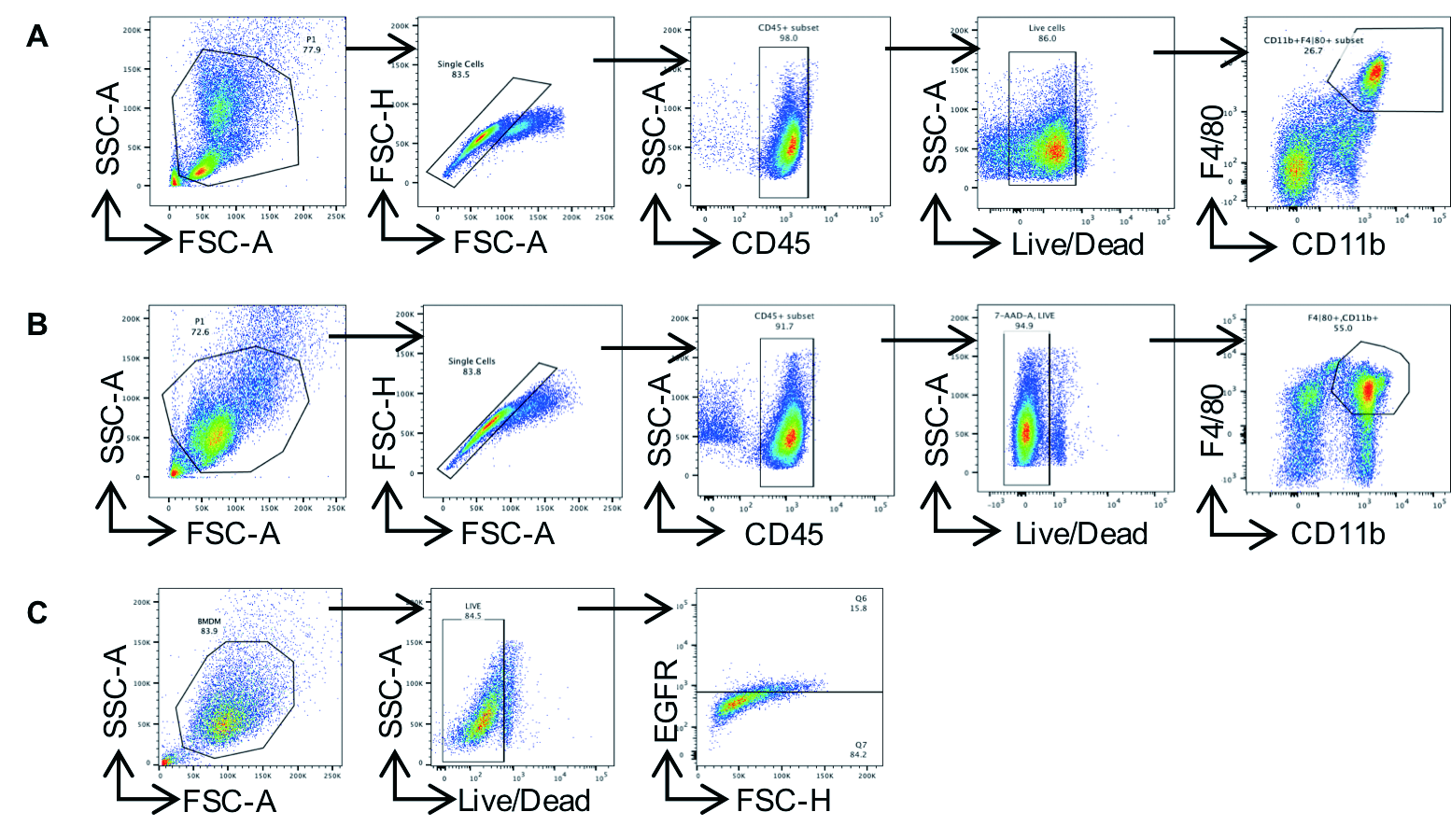

Supplement: Supplementary file 6 — Figure S6 [file 41419_2022_5370_MOESM6_ESM.tif]
