# Supplementary material for: EGFR tyrosine kinase activity and Rab GTPases coordinate EGFR trafficking to regulate macrophage activation in sepsis
Source: Cell Death Dis. 2022 Nov 7;13(11):934. doi: 10.1038/s41419-022-05370-y (PMC9640671; doi:10.1038/s41419-022-05370-y)
Supplement: Supplementary file 7 — Supplementary Figure legends [file 41419_2022_5370_MOESM7_ESM.docx]

**Supplementary Figure legends**

**Fig S1. LPS induces phosphorylation of Rab7a at serine 72.** **A** Number of identified phosphoSites, phosphopeptides, phosphoProteins. The complete list of identified proteins is shown in Table S1. **B** Most enriched functions associated with LPS-induced phosphoProteins (FDR < 0.05). The complete list of identified proteins is shown in Table S2. (**C-D**) Volcano plot of –log (P value) versus the log2FC (fold change) for quantified phosphopeptides for RAW264.7 were treated with LPS (1 μg/mL) for 30min with or without PD168393(PD) pretreatment for 30min. Phosphorylation site and number of peptides quantified are shown in parenthesis. The complete list of identified phosphopeptide is shown in Table S3 and Table S4.

**Fig S2. EGFR phosphorylation promotes M1 macrophage polarization.** (**A-F**) RAW264.7 cells or MH-S cells were treated with LPS (1μg/mL) for 24 h with or without PD168393(10μM) pretreatment for 30min. A Real time PCR analysis of IL-1β and iNOS expression in RAW264.7 (n=3). **B** Western blot analysis of iNOS in RAW264.7 cell lysates, a-β-actin as a loading control. **C** Flow cytometry analysis the level of M1 macrophage-associated markers iNOS in RAW264.7. **D** Percentage and MFI of iNOS-positive cells are shown in RAW264.7 (n=3). **E** Flow cytometry analysis the level of M1 macrophage-associated markers iNOS in RAW264.7. **F** Percentage and MFI of iNOS-positive cells is shown in RAW264.7 (n=3). The graphs depict mean ± SD based on three independent experiments. *P<0.05, **P < 0.01, ***P < 0.001, ns stands for non-significant difference.

**Fig. S3. Inhibition of EGFR phosphorylation promoted M2 macrophage polarization.** (**A-G**) Murine alveolar macrophage cell line MH-S cells were treated with LPS (1μg/mL) for 24h with or without Erlotinib (10μM) pretreatment for 30min or Rosiglitazone (Rosi (20μM) pretreatment for 24h. A RT-qPCR analysis of mRNA expression of M2-related genes Mcr1 (n = 3). **B** Western blot analysis of ARG1, a-Tubulin as a loading control. **C** Flow cytometry analysis of the level of M2 macrophage-associated markers (CD206). **D** Percentage and MFI of CD206-positive cells is shown (n=3). **E** Real time PCR analysis of ARG1 expression (n=3). **F** Real time PCR analysis of Mcr-1 expression (n=3). **G** Real time PCR analysis of Ym1 expression (n=3). The graphs depict mean ± SD based on three independent experiments. *P<0.05, **P < 0.01, ***P < 0.001, ns stands for non-significant difference.

**Fig. S4.** **LPS induces macrophage M1/M2 polarization is EGFR ligand-independent.** (**A-F**) BMDM were treated with LPS (1 μg/mL) 24 h in the presence or absence of pretreatment of mouse anti-EGF (20 ng/mL) for 30 min. **A** Western blot analysis of EGFR in whole-cell lysates of BMDM, a-Tubulin as a loading control, and Western blot analysis of EGF in cell supernatants, proteins from cell supernatants were extracted by methanol–chloroform precipitation. **B** Real time PCR analysis of IL-1β expression (n=3). **C** Real time PCR analysis of iNOS expression (n=3). **D** Flow cytometry analysis of cell surface iNOS. **E** Percentage of iNOS-positive BMDM is shown (n=3). **F** Mean fluorescence intensity (MFI) of iNOS is shown (n = 3). (**G**-**K**) BMDM were treated with LPS (1 μg/mL) 24 h in the presence or absence of pretreatment of mouse anti-EGF (20 ng/mL) / PD168393 (PD, 10 μM) for 30 min. G Real time PCR analysis of Mcr-1 expression (n=3). **H** Real time PCR analysis of Ym1 expression (n=3). **I** Flow cytometry analysis of cell surface CD206. **J** Percentage of CD206-positive BMDM is shown (n=3). **K** Mean fluorescence intensity (MFI) of CD206 is shown (n = 3). The graphs depict mean ± SD based on three independent experiments. *P<0.05, **P < 0.01, ***P < 0.001, ns stands for non-significant difference.

**Fig. S5. Deletion of EGFR inhibits LPS induces M1 polarization and potentiates M2 polarization in macrophages.**

A PCR analysis showing efficient EGFR recombination in BMDM from EGFRflox/flox and EGFR-KO (LysM-Cre; EGFRflox/flox) mice. B Western blot analysis of EGFR, a-GAPDH as a loading control. (C-L) WT and EGFR−/− BMDM were treated with LPS (1 μg/mL) for 24 h. C Real time PCR analysis of IL-1β expression (n=3). D Real time PCR analysis of iNOS expression (n=3). E Real time PCR analysis of Mcr-1 expression (n=3). F Real time PCR analysis of Ym1 expression (n=3). G Flow cytometry analysis of cell surface iNOS. H Percentage of iNOS-positive BMDM is shown (n=3). I Mean fluorescence intensity (MFI) of iNOS is shown (n = 3). J Flow cytometry analysis of cell surface CD206. K Percentage of CD206-positive BMDM is shown (n=3). L Mean fluorescence intensity (MFI) of CD206 is shown (n = 3). The graphs depict mean ± SD based on three independent experiments. *P<0.05, **P < 0.01, ***P < 0.001.

**Fig. S6.** **Phenotypic characterization of macrophages by flow cytometry.**

(A-C) Example flow spectrometry gating strategy for (A) peritoneal macrophages, (B) alveolar macrophages, (c) BMDM. Dead cells were excluded by 7-AAD staining.
