# Supplementary material for: EGFR tyrosine kinase activity and Rab GTPases coordinate EGFR trafficking to regulate macrophage activation in sepsis
Source: Cell Death Dis. 2022 Nov 7;13(11):934. doi: 10.1038/s41419-022-05370-y (PMC9640671; doi:10.1038/s41419-022-05370-y)
Supplement: Supplementary file 8 — Full uncut gels [file 41419_2022_5370_MOESM8_ESM.pdf]

Supplementary Figs | Uncropped western blot images of the indicated Figures.

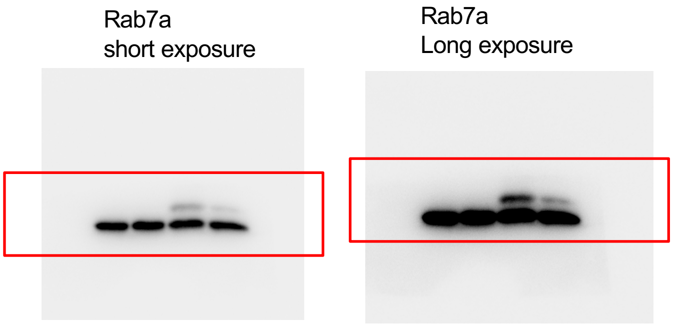

Full unedited gel for Figure 2B

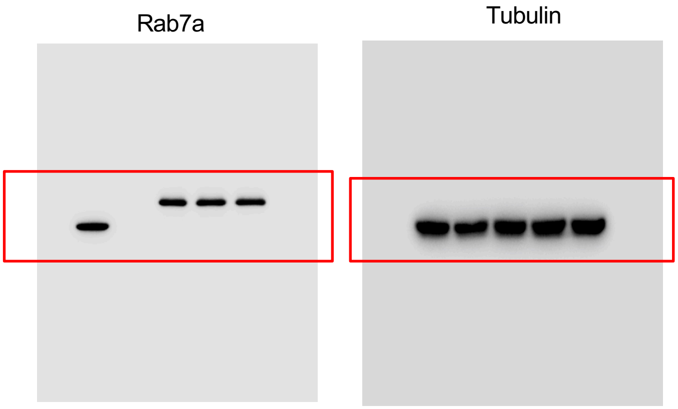

Full unedited gel for Figure 2c

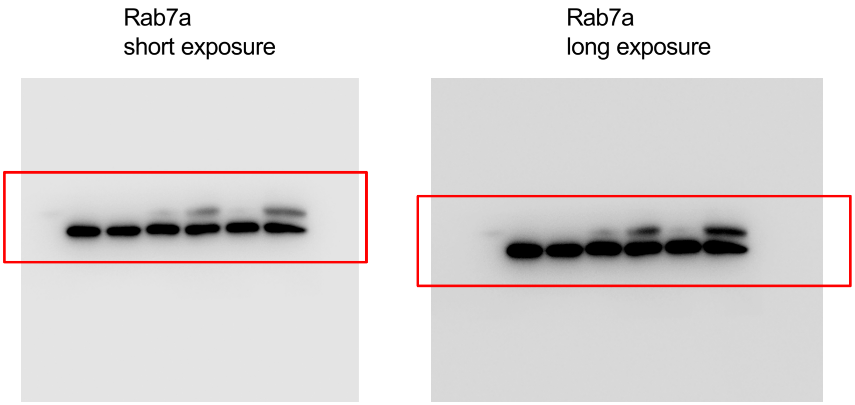

Full unedited gel for Figure 2D

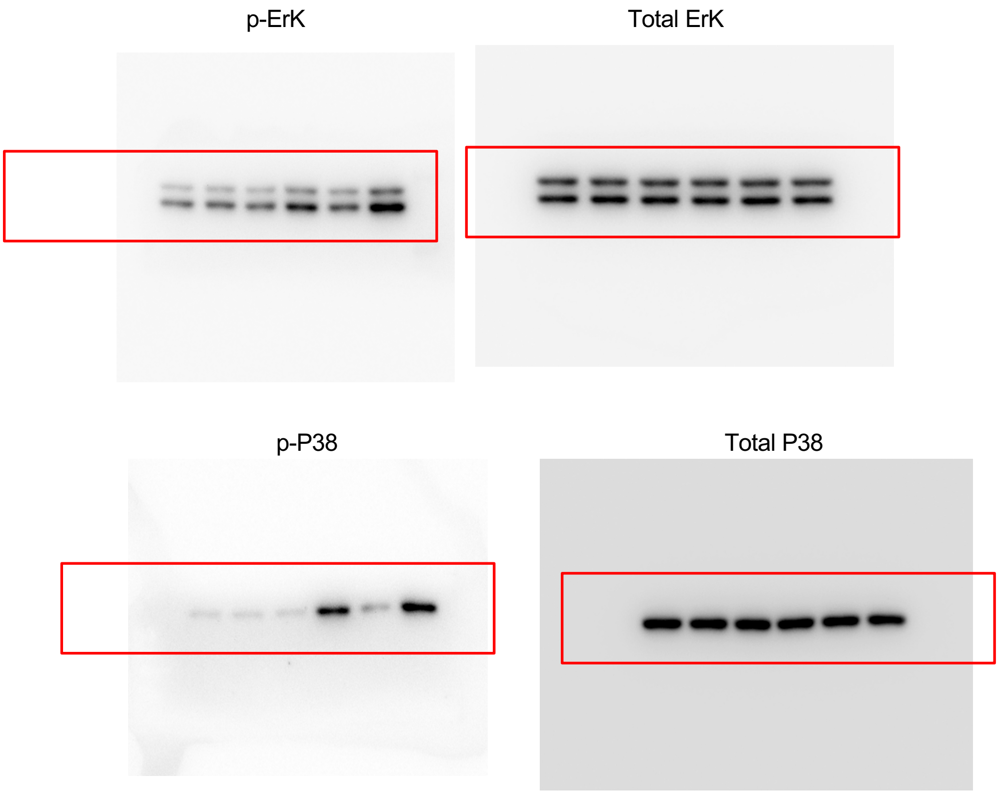

Full unedited gel for Figure 2J

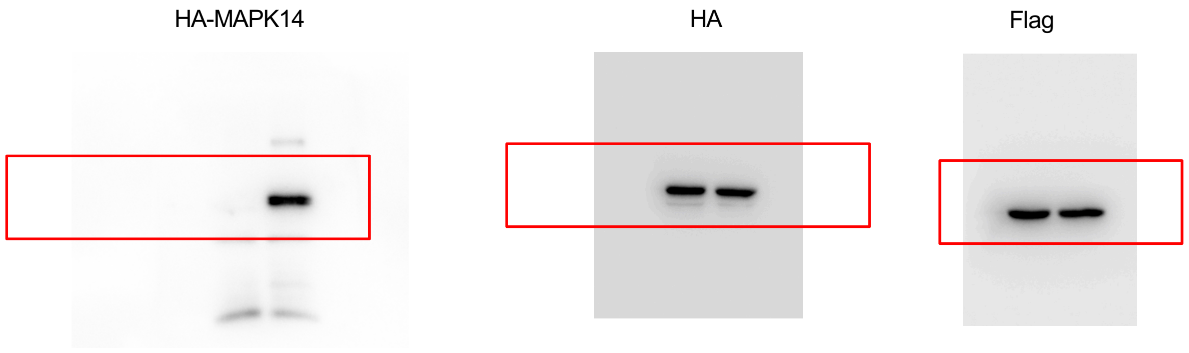

Full unedited gel for Figure 3B

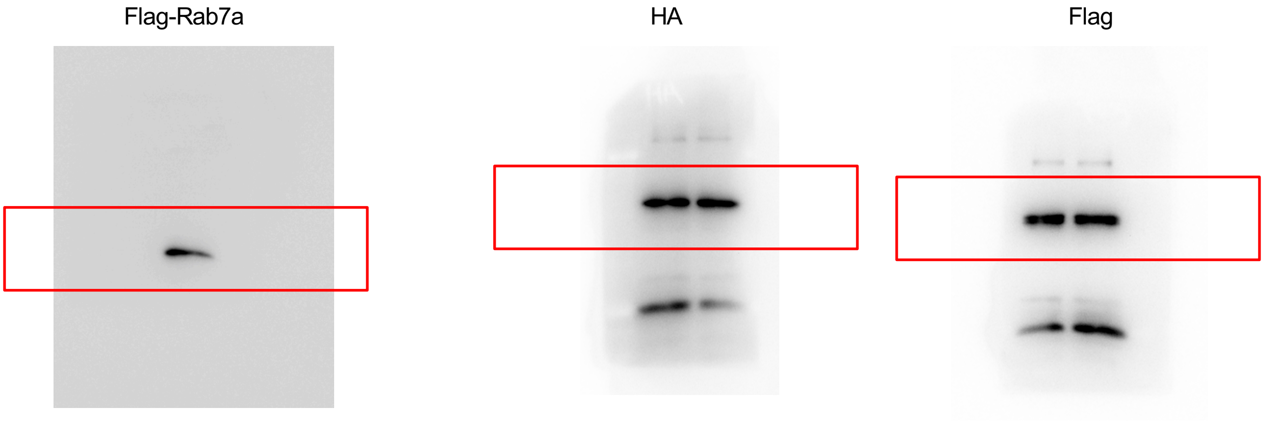

Full unedited gel for Figure 3C

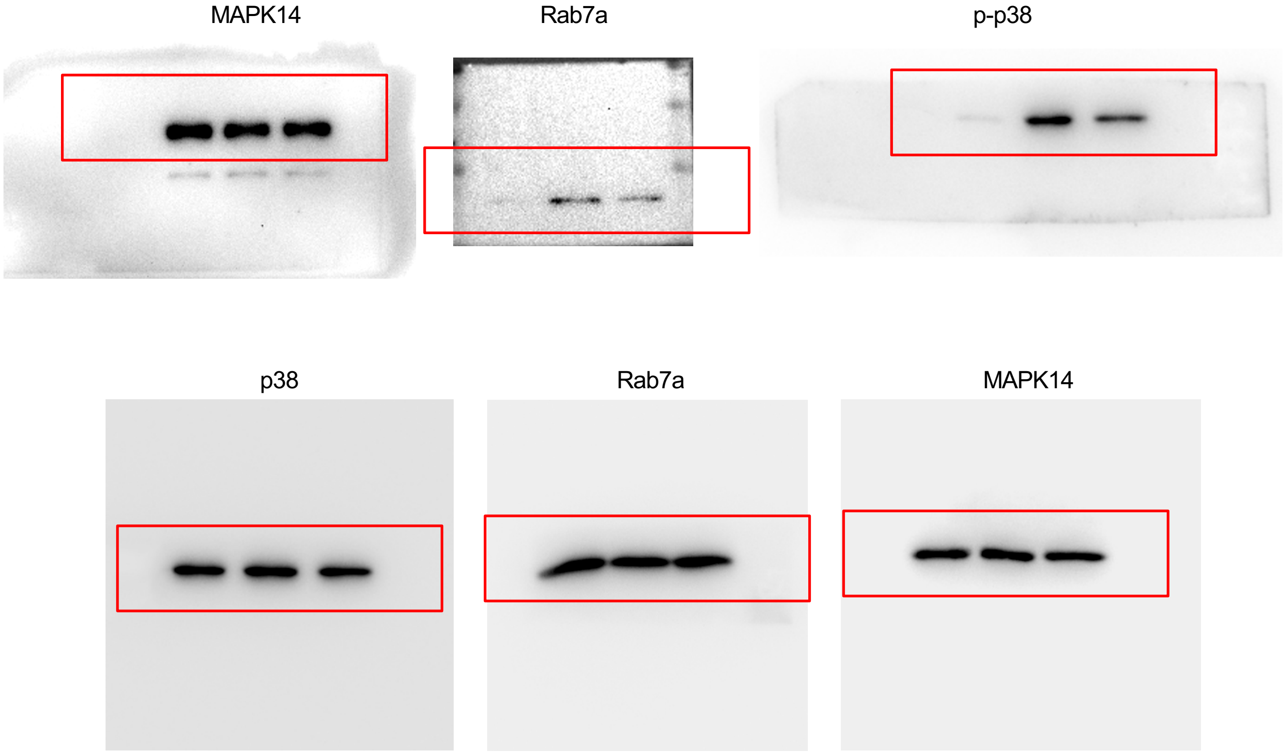

Full unedited gel for Figure 3D

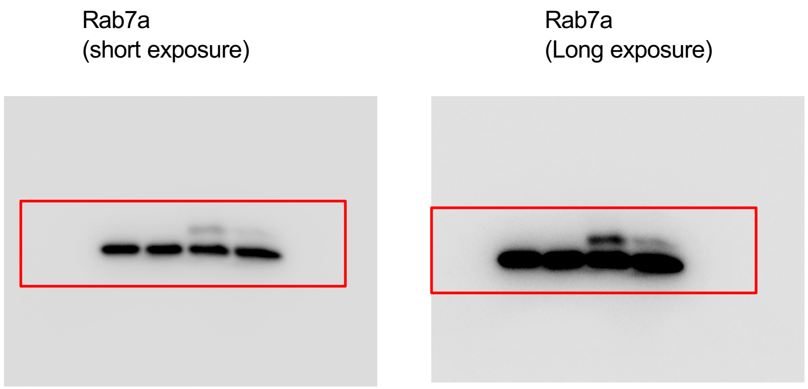

Full unedited gel for Figure 3E

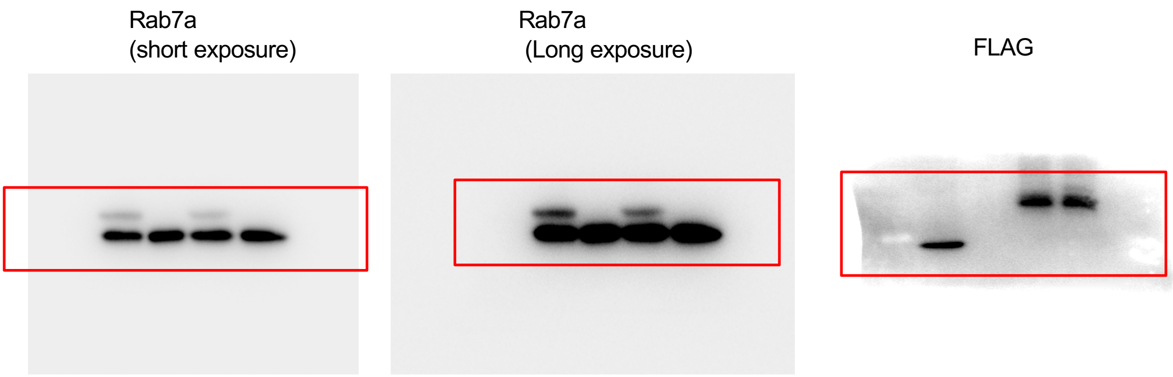

Full unedited gel for Figure 3F

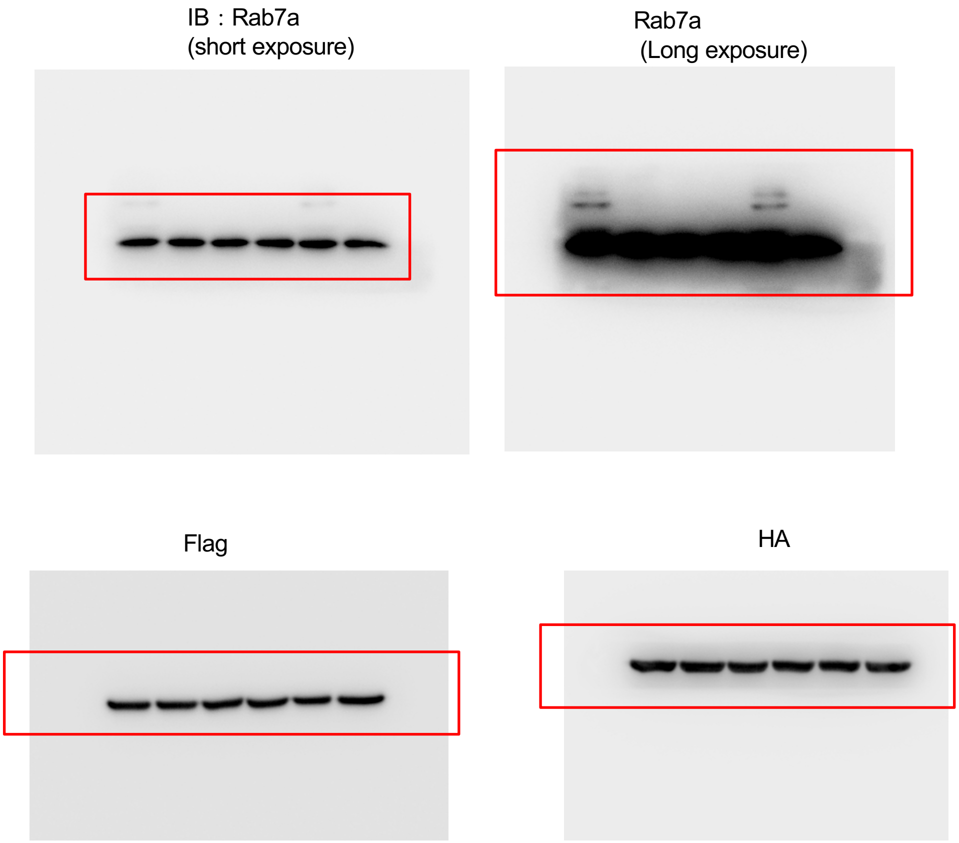

Full unedited gel for Figure 3G

EGFR

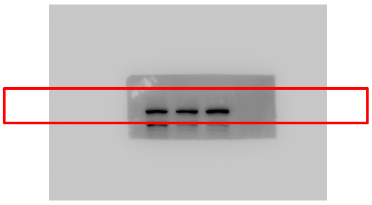

Rab5a

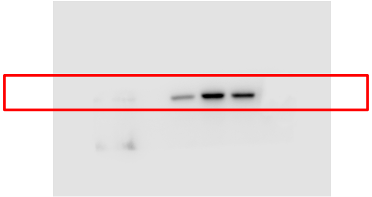

Full unedited gel for Figure 4C

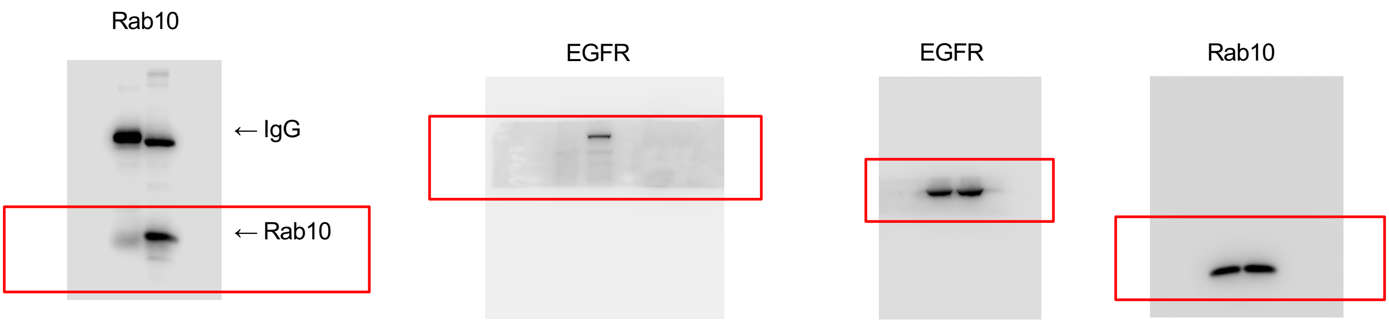

Full unedited gel for Figure 5C

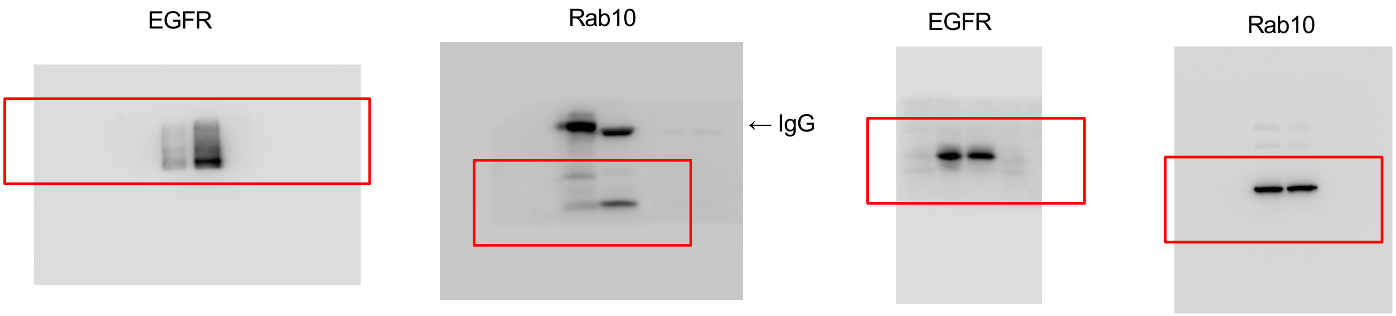

Full unedited gel for Figure 5D

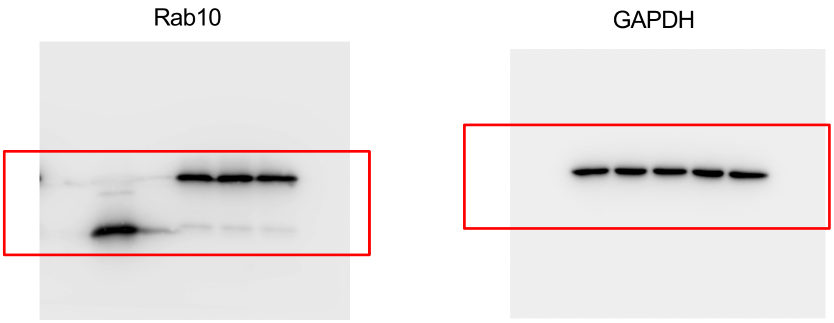

Full unedited gel for Figure 5G

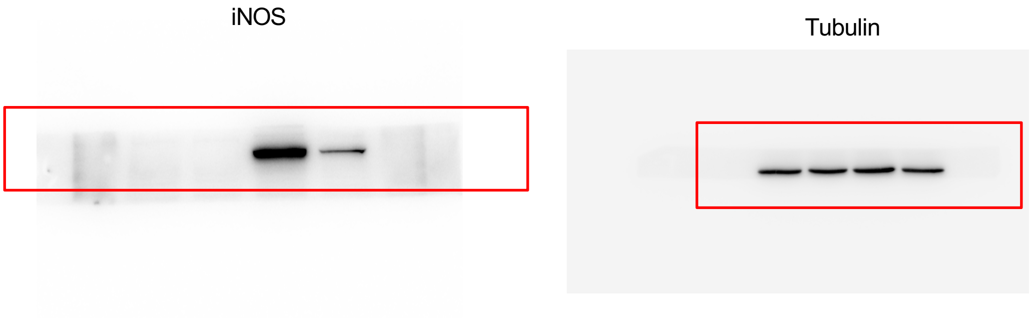

Full unedited gel for Figure 6B

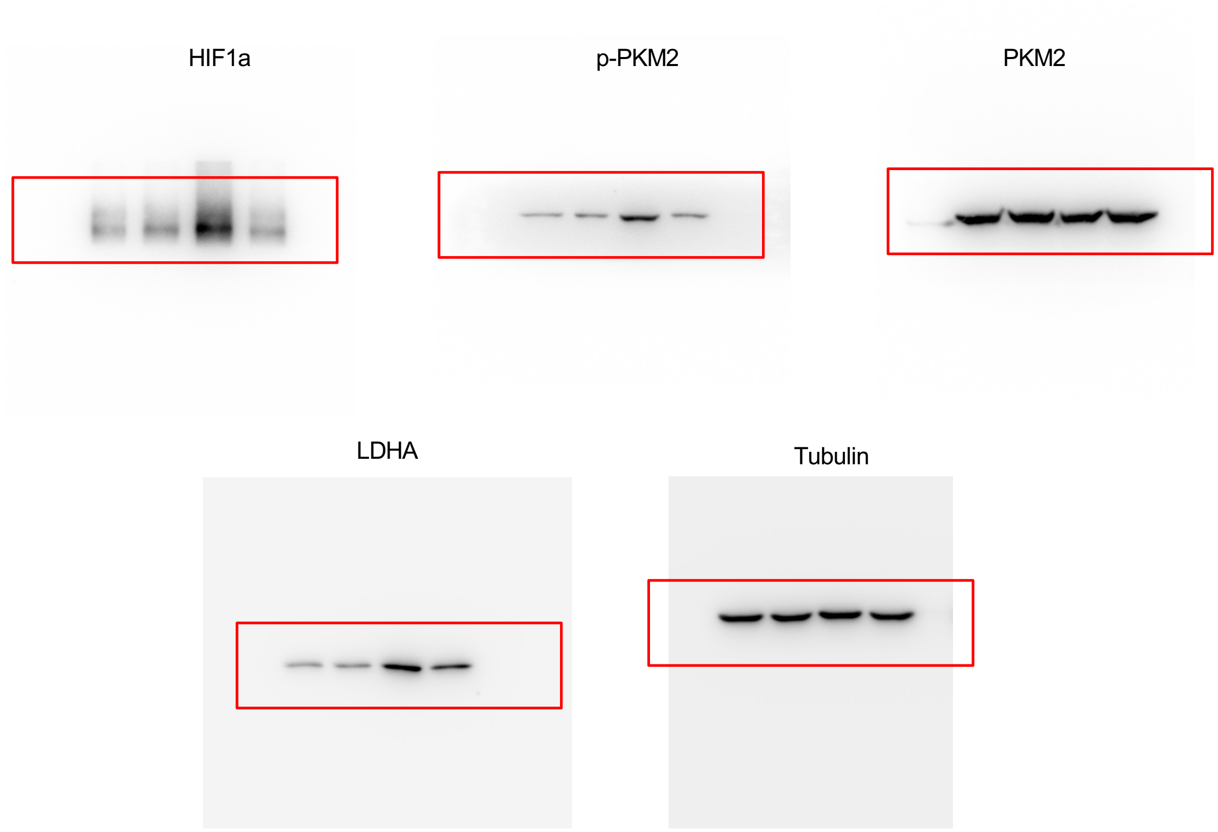

Full unedited gel for Figure 6M

Fig. 7

Arg1

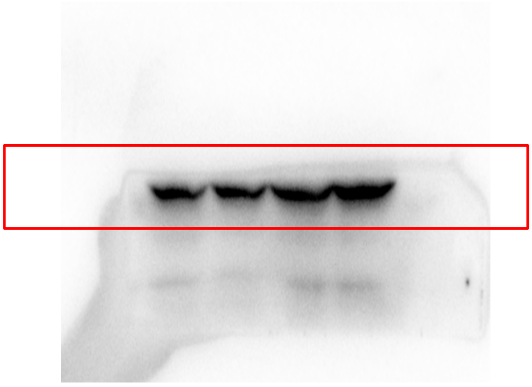

Tubulin

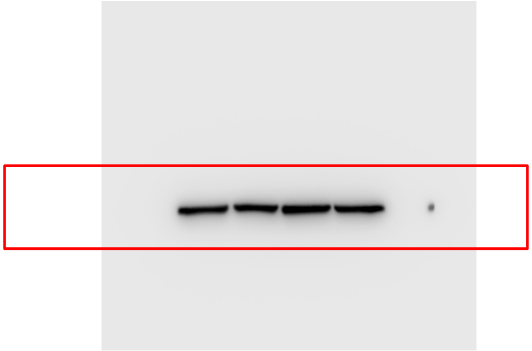

Full unedited gel for Figure 7C

p-PPARγ
(Ser112)

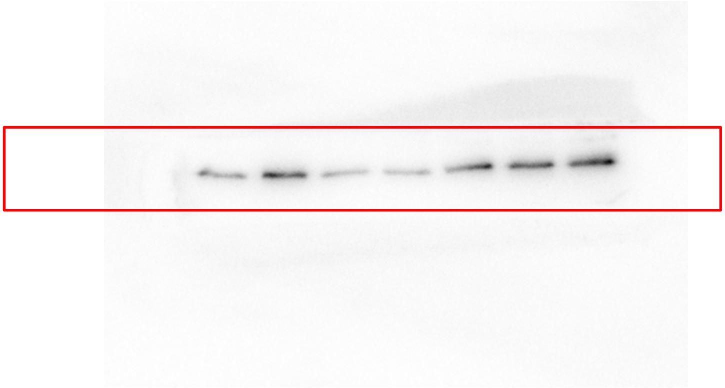

t-PPARγ

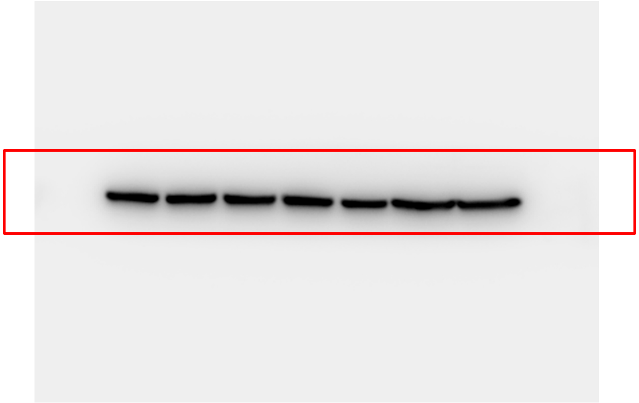

GAPDH

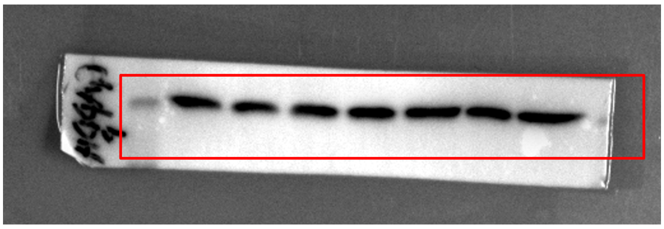

Full unedited gel for Figure 7J

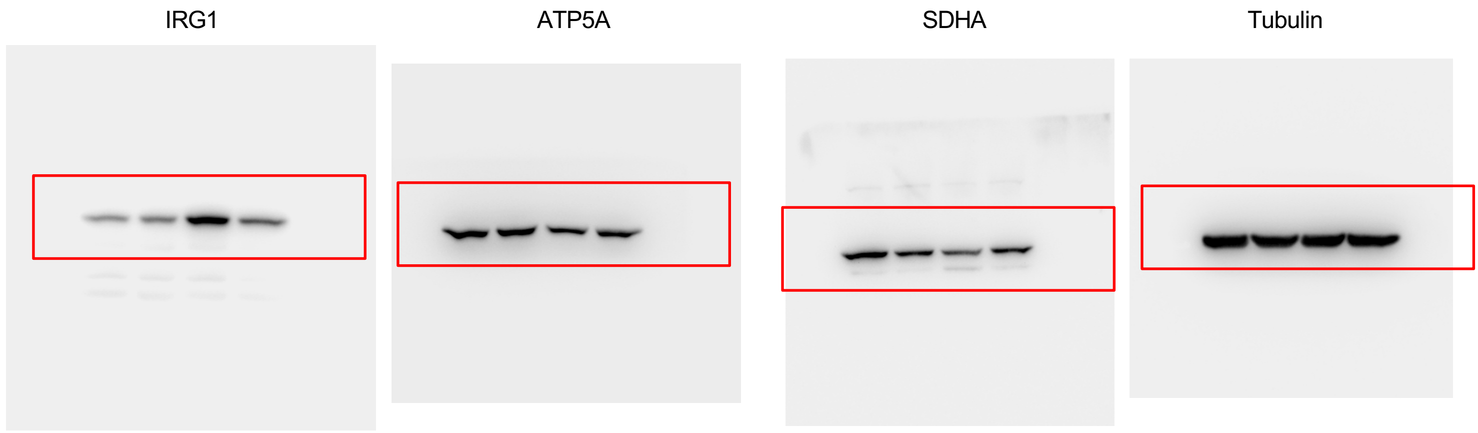

Full unedited gel for Figure 7U

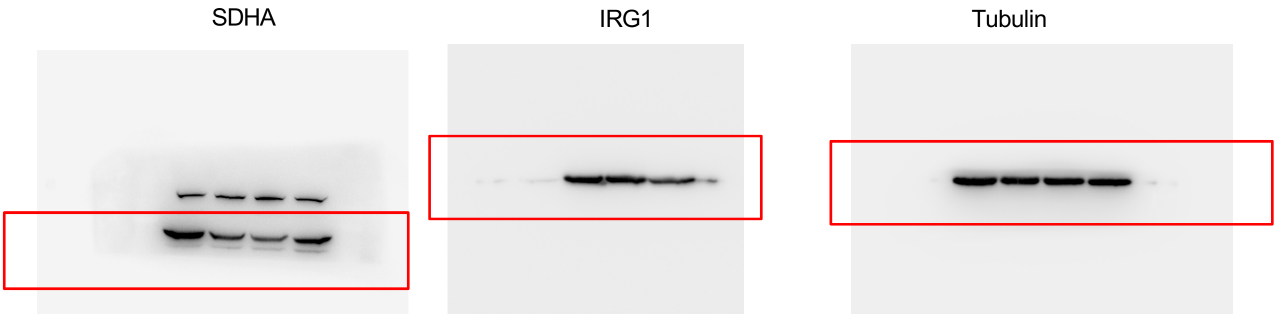

Full unedited gel for Figure 7V

iNOS

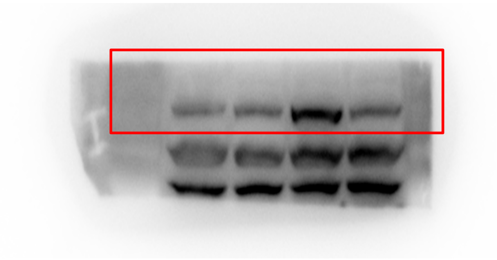

β-actin

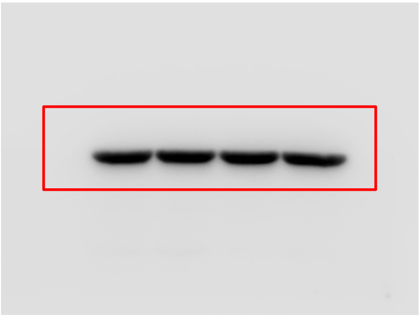

Full unedited gel for Figure S2B

Arg1

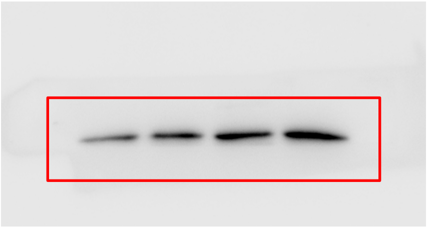

Tubulin

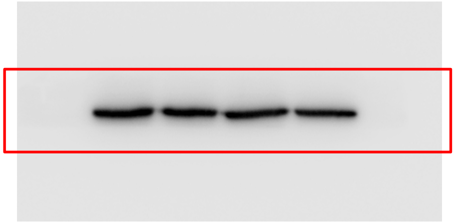

Full unedited gel for Figure S3B

iNOS

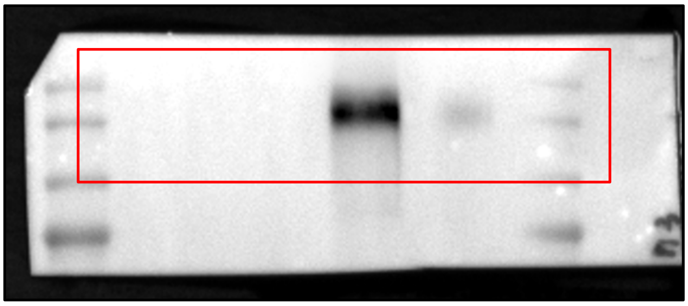

β-actin

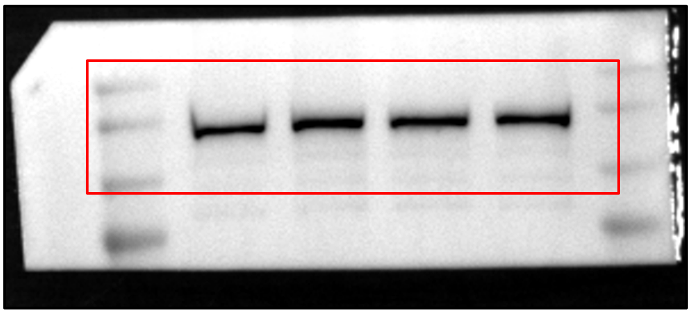

Tubulin

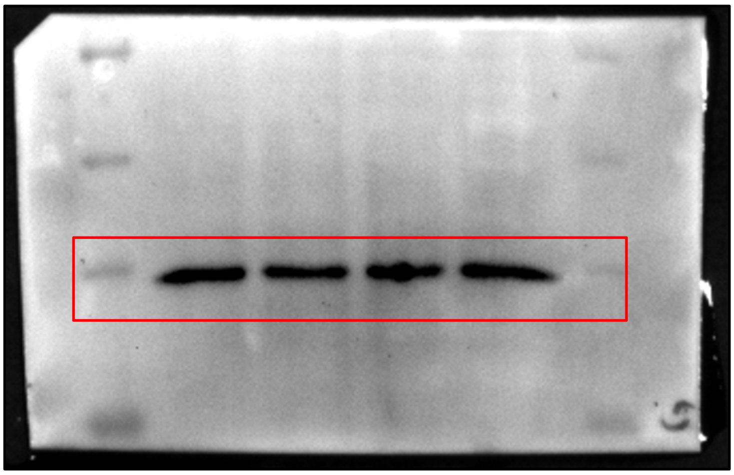

Full unedited gel for Figure S4A

EGFR

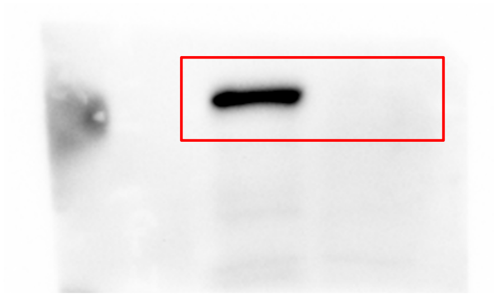

Tubulin

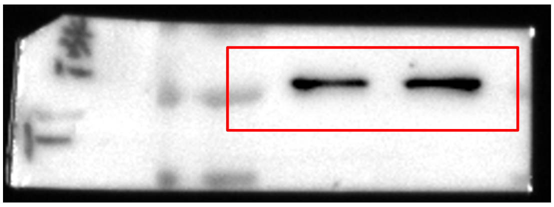

Full unedited gel for **Fig S5**B
