# Supplementary material for: EGFR tyrosine kinase activity and Rab GTPases coordinate EGFR trafficking to regulate macrophage activation in sepsis
Source: Cell Death Dis. 2022 Nov 7;13(11):934. doi: 10.1038/s41419-022-05370-y (PMC9640671; doi:10.1038/s41419-022-05370-y)
Supplement: Supplementary file 15 — Study approval [file 41419_2022_5370_MOESM15_ESM.pdf]

研究进展报告

项目	EGFR通过磷酸化RAB7A介导代谢重编程调控巨噬细胞极化在脓毒症中的作用及分子机制研究		
项目来源	自筹经费		
方案版本号	1.0	方案版本日期	2020年09月28日
知情同意书版本号	1.0	知情同意书版本日期	2020年09月28日
伦理审查批件号	PJ2020-077	本院主要研究者	唐靖
伦理审查批件有效期	2020-10-15~2022-10-30		
本次报告类型	■定期报告 □年度报告		

一、受试者信息

- 合同研究总例数： 50
- 已入组例数： 33
- 完成观察例数： 33
- 提前退出例数： 0
- 严重不良事件例数： 0
- 已报告的严重不良事件例数： 0

二、研究情况

- 研究阶段： □研究尚未启动， □正在招募受试者（尚未入组）， ■正在实施研究， □受试者的试验干预已经完成， □后期数据处理阶段
- 是否存在影响研究进行的情况： ■否， □是→请说明：
- 是否存在与试验干预相关的、非预期的、严重不良事件： □是， ■否
- 研究风险是否超过预期： □是， ■否
- 是否存在影响研究风险与受益的任何新信息、新进展： ■否， □是→请说明：
- 研究中是否存在影响受试者权益的问题： ■否， □是→请说明：
- 严重不良事件或方案规定必须报告的重要医学事件已经及时报告: ■不适用, □是 □否

三、其它

- 是否申请延长伦理审查批件的有效期： □是 ■否（选择“是”，填写下列选项）
  - ✧ 申请延长至的日期：
  - ✧ 申请延长的理由：

申请人签字	唐靖	日期	2021年11月03日
