# Supplementary material for: EGFR tyrosine kinase activity and Rab GTPases coordinate EGFR trafficking to regulate macrophage activation in sepsis
Source: Cell Death Dis. 2022 Nov 7;13(11):934. doi: 10.1038/s41419-022-05370-y (PMC9640671; doi:10.1038/s41419-022-05370-y)
Supplement: Supplementary file 16 — ClinicalTrials identifier [file 41419_2022_5370_MOESM16_ESM.pdf]

审核状态：<br>Project audit state：	通过审核<br>Successful
注册号：<br>Registration number：	ChiCTR2000039545
最近更新时间：<br>Date of Last Refreshed on：	2020/10/31 3:39:46
注册号状态：	预注册
Registration Status：	Prospective registration
填写语言：	中文和英文
Language：	Chinese And English
注册题目：	EGFR通过磷酸化RAB7A介导代谢重编程调控巨噬细胞极化在脓毒症中的作用及分子机制研究
Public title：	EGFR regulates macrophage polarization by phosphorylating Rab7A-mediated metabolic reprogramming Study on the role and molecular mechanism of sepsis
注册题目简写：	
Public title acronym：	
研究课题的正式科学名称：	EGFR通过磷酸化RAB7A介导代谢重编程调控巨噬细胞极化在脓毒症中的作用及分子机制研究
Scientific title：	EGFR regulates macrophage polarization by phosphorylating Rab7A-mediated metabolic reprogramming Study on the role and molecular mechanism of sepsis
研究课题的正式科学名称简写：	
Scientific title acronym：	
研究课题代号(代码)：<br>Study subject ID：	
在其它机构的注册号：<br>Secondary ID：	

申请注册联系人：	陈翠平	研究负责人：	唐靖
Applicant：	chen cui ping	Study leader：	Tang Jing
申请注册联系人电话：<br>Applicant telephone：	13729016162	研究负责人电话：<br>Study leader's telephone：	13189091282
申请注册联系人传真 ：<br>Applicant Fax：		研究负责人传真：<br>Study leader's fax：	
申请注册联系人电子邮件：<br>Applicant E-mail：	271783511@qq.com	研究负责人电子邮件：<br>Study leader's E-mail：	tanglitangjing@126.com
申请单位网址(自愿提供)：<br>Applicant website(voluntary supply)：		研究负责人网址(自愿提供)：<br>Study leader's website(voluntary supply)：	
申请注册联系人通讯地址：	广东省湛江市人民大道南57号	研究负责人通讯地址：	广东省湛江市人民大道南57号
Applicant address：	57 Renmin Dadao South, Zhanjiang City, Guangdong Province, China	Study leader's address：	57 Renmin Dadao South, Zhanjiang City, Guangdong Province, China
申请注册联系人邮政编码：<br>Applicant postcode：		研究负责人邮政编码：<br>Study leader's postcode：	
申请人所在单位：	广东医科大学附属医院		
Applicant's institution：	Affiliated Hospital of Guangdong Medical University		

是否获伦理委员会批准：	是		
Approved by ethic committee：	Yes		
伦理委员会批件文号：<br>Approved No. of ethic committee：	PJ2020-077	伦理委员会批件附件：<br>Approved file of Ethical Committee：	查看附件View
批准本研究的伦理委员会名称：	广东医科大学附属医院机构审查伦理委员会		
Name of the ethic committee：	Review Ethics Committee of Affiliated Hospital of Guangdong Medical University		
伦理委员会批准日期：<br>Date of approved by ethic committee：			
国家FDA批准文号：<br>Approved No. of SFDA：		国家FDA批准附件：<br>Approved file of SFDA：	
国家FDA批准日期：<br>Date of approved by SFDA：			

研究实施负责（组长）单位： 广东医科大学附属医院

Primary sponsor:	Affiliated Hospital of Guangdong Medical University
研究实施负责（组长）单位地址:	广东省湛江市人民大道南57号
Primary sponsor's address:	57 Renmin Dadao South, Zhanjiang City, Guangdong Province, China

试验主办单位(项目批准或申办者): Secondary sponsor:

国家:	中国	省(直辖市):	广东	市(区县):	湛江
Country:	CHINA	Province:	Guangdong	City:	Zhanjiang
单位(医院):	广东医科大学附属医院	具体地址:	人民大道南57号		
Institution hospital:	Affiliated Hospital of Guangdong Medical University	Address:	57 Renmin Avenue South		

经费或物资来源:	自筹经费
Source(s) of funding:	self-financing
研究疾病:	脓毒症
Target disease:	Sepsis
研究疾病代码:	
Target disease code:	
研究类型:	基础科学研究
Study type:	Basic Science
研究所处阶段:	其它
Study phase:	N/A
研究目的:	探讨EGFR膜定位在脓毒症巨噬细胞活化中的作用，以及调控EGFR膜定位的分子机制。
Objectives of Study:	To investigate the role of EGFR membrane localization in the activation of sepsis macrophages and the molecular mechanism of regulating EGFR membrane localization.
研究设计:	病例对照研究
Study design:	Case-Control study
纳入标准:	①患者或家属对研究知情同意并签署知情同意书 ②住院脓毒症患者 ③男女不限，年龄18周岁以上 ④无其他严重系统疾病的患者
Inclusion criteria	①Patients or family members give informed consent to the study and sign the informed consent ②Hospitalized patients with sepsis ③No male or female, age 18 or above ④Patients without other serious systemic diseases
排除标准:	①伴有严重心肝肾和造血系统疾病 ②合并恶性肿瘤患者 ③术前患有中枢神经系统和心理疾病
Exclusion criteria:	①Accompanied by severe heart, liver, kidney and hematopoietic diseases ②Patients with complicated malignant tumor ③He had a central nervous system and mental illness before surgery
研究实施时间: Study execute time:	从From2020-10-10至To 2021-10-10

干预措施: Interventions:

组别:	非脓毒症患者组和脓毒症患者组	样本量:	50
Group:	Group of patients with sepsis	Sample size:	
干预措施:	抽取脓毒症患者的血液	干预措施代码:	
Intervention:	Collect blood from patients with sepsis	Intervention code:	

研究实施地点: Countries of recruitment and research settings:

国家:	中国	省(直辖市):	广东	市(区县):	
Country:	China	Province:	Guangdong	City:	
单位(医院):	广东医科大学附属医院	单位级别:	三级甲等		
Institution hospital:	Affiliated Hospital of Guangdong Medical University	Level of the institution:	Tertiary A		

测量指标: Outcomes:

指标中文名:	表皮生长因子	指标类型:	主要指标
Outcome:	epidermal growth factor	Type:	Primary indicator
测量时间点:		测量方法:	
Measure time point of outcome:		Measure method:	

采集人体标本: Collecting sample(s) from participants:

标本中文名:	血液	组织:	
Sample Name:	blood	Tissue:	
人体标本去向	使用后销毁	说明	
Fate of sample:	Destruction after use	Note:	

征募研究对象情况：<br>Recruiting status：	尚未开始<br>Pending
年龄范围：<br>Participant age：	最小 Min age 18 岁 years<br>最大 Max age 80 岁 years
性别：	男女均可
Gender：	Both
随机方法（请说明由何人用什么方法产生随机序列）：	由护士采取真空取血法抽取脓毒症患者的血液
Randomization Procedure (please state who generates the random number sequence and by what method)：	The blood of patients with sepsis is drawn by the nurse by vacuum.
UTN(全球唯一识别码)：	
盲法：	
Blinding：	
试验完成后的统计结果：	
Calculated Results ater the Study Completed：	
研究负责(组长)单位：	
Organizer institution (leader institution)：	
资料收集汇总单位：	0
Data collection Institution：	
资料管理单位：	项目组根据研究就进程择期选择具体公开原始数据
Data management Institution：	According to the research process, the project team selects the specific release of the original data.
资料分析单位：	研究者根据受试者的原始观察记录，将数据及时、完整、正确、清晰地输入病例报告表。录入采用相同的数据库系统双人双机录入，之后对数据库进行两遍比对。电子数据文件分类保存，并有多个备份保存于不同磁盘或记录介质上，妥善保存，禁止破坏。
Data analysis Institution：	According to the original observation records of the subjects, the researcher will input the data into the case report form timely, complete, correct and clear.The same database system was used for input, and then the database was compared twice.Electronic data files are stored separately and with multiple backups on different disks or recording media. They are properly stored and no damage is allowed.
